# Serendipitous Hinge Modulation Hypothetically Reprograms Caerin 1.1-LC Antibacterial Mechanism and Gram-Negative Selectivity

**DOI:** 10.3390/pharmaceutics17111500

**Published:** 2025-11-20

**Authors:** Zhengze Sun, Ruixin Zhao, Yueao Zhang, Xiaonan Ma, Yangyang Jiang, Tao Wang, Xiaoling Chen, Chengbang Ma, Tianbao Chen, Chris Shaw, Mei Zhou, Lei Wang

**Affiliations:** 1 Natural Drug Discovery Group, School of Pharmacy, Queen’s University Belfast, Belfast BT9 7BL, UK; 2 Department of Surgical Oncology, The First Affiliated Hospital of China Medical University, Shenyang 110001, China

**Keywords:** Caerin, hinge structure, antimicrobial peptides, mechanism of action, drug resistance

## Abstract

**Background:** The golden era of antibiotics has long passed, and the clinical failures caused by emerging drug-resistant bacteria have intensified the demand for novel antimicrobial agents. Antimicrobial peptides have attracted significant attention as promising candidates for next-generation antibiotics. **Methods:** In this study, we identified a novel antimicrobial peptide, Caerin 1.1-LC, from the skin secretion of the Australian green tree frog, *Litoria caerulea*. Subsequent structure–activity relationship studies led us to design a series of analogues and revealed the critical role of the peptide’s intrinsic hinge structure in shaping its biological activity. **Results:** Incorporation of D-isomers at the valine residues within the hinge preserved overall helical content but altered the hinge conformation, resulting in an 8-fold increase in antibacterial activity against Gram-negative bacteria. Simultaneously, haemolytic activity was markedly reduced, leading to a 56-fold improvement in therapeutic index (from 0.47 to 26.6). Structural modulation of the hinge also switched the mechanism of action from classical membrane disruption with associated permeability changes to a non-membrane-permeabilising, ‘cell-penetrating-like’ behaviour, inducing membrane potential depolarisation and ATP disruption to trigger bacterial death. In vivo studies using infected larval models, along with in vitro LPS neutralisation assays, further demonstrated the therapeutic potential of the D-analogue as a novel antibacterial agent. **Conclusions:** This work highlights the pivotal role of hinge structures in Caerin-family/hinge-containing AMPs, offering a strategic avenue for optimising antibacterial efficacy.

## 1. Introduction

Over the past decade, increasing concerns over drug resistance have prompted efforts to discover novel antimicrobial agents [1,2,3,4]. Antimicrobial peptides (AMPs) are considered promising candidates for next-generation antibiotics due to their broad spectrum and low tendency to induce resistance [5,6,7]. However, naturally occurring AMPs often face challenges for clinical use, including poor stability, low antibacterial activity and high cytotoxicity [8,9,10]. Consequently, a clear strategy is required to modify AMPs from amphibian skin secretions to meet clinical requirements.

Caerin 1 peptides, part of the Caerin AMP family, are derived from the skin secretions of Australian tree frogs of the genus *Litoria* [11]. These peptides typically feature two α-helices separated by a hinge region containing two proline residues. This structure facilitates a carpet-model mechanism for membrane disruption [12]. The hinge structure provides flexibility, allowing the peptide to adjust conformationally after binding to the bacterial membrane [13,14]. This flexibility increases the contact surface area, enhancing membrane permeability disruption [15]. Additionally, the introduction of a hinge structure can reduce cytotoxicity. For instance, the hinge structure in lsCT enhances its selectivity and reduces cytotoxicity [16]. Conversely, deleting the hinge in Brevinin-1Bya increases its cytotoxicity due to reduced structural flexibility [17]. Therefore, the hinge structure plays a crucial role in regulating both antimicrobial activity and cytotoxicity, a role further explored in this study for Caerin 1 peptides.

In this work, a novel Caerin 1 peptide, Caerin 1.1-LC, was identified from *Litoria caerulea* skin secretions. Structure–activity relationship studies led to the development of a set of analogues designed to investigate the helix sections and hinge structure. Notably, the introduction of D-isomers into the hinge region revealed their significant role in modulating antimicrobial activity. The presence of D-isomers not only decreased the cytotoxicity of the Caerin 1.1 V(D)V(D) against healthy cells, increasing selectivity, especially against Gram-negative bacteria, but also altered its antibacterial mechanism. Mechanism studies indicated that the D-isomer does not induce the classic membrane disruption typically seen with amphibian AMPs. Instead, it enables the peptide to penetrate the bacterial membrane, causing membrane depolarisation and disrupting ATP production. Additionally, these peptides exhibited comparable inhibition of pro-inflammatory cytokine production in LPS-stimulated murine macrophage models. The findings in this study not only highlight the critical role of the hinge structure in bacterial killing of Caerin 1 peptides but also demonstrate the development of a novel ‘cell-penetrating peptide (CPP)-like’ AMP, which may serve as a carrier or vehicle for functional motifs or antibiotics, paving the way for the design of specifically targeted AMPs (STAMPs) or antibacterial conjugates with promising therapeutic potential.

## 2. Materials and Methods

### 2.1. Collections of Skin Secretions from Litoria Caerulea

*Litoria caerulea* (*L. caerulea*) specimens were obtained from commercial sources and maintained at Queen’s University Belfast for three months prior to the collection of skin secretions. Secretions were obtained using mild transdermal electrical stimulation, as previously described, then lyophilised and stored at −20 °C until molecular cloning was performed. Collection procedures were carried out by Dr. Mei Zhou in accordance with the United Kingdom Animals (Scientific Procedures) Act 1986 under project license PPL 2800, issued by the Department of Health, Social Services and Public Safety, Northern Ireland. All procedures were reviewed by the Institutional Animal Care and Use Committee (IACUC) of Queen’s University Belfast and approved on 19 February 2016.

### 2.2. Molecular Cloning

Five milligrams of lyophilised skin secretion from *Litoria caerulea* was used for mRNA isolation. Polyadenylated mRNA was isolated using the mRNA Purification Kit (Invitrogen, Carlsbad, CA, USA). Subsequently, a cDNA library was constructed with the SMARTer^®^ Rapid Amplification of cDNA Ends (RACE) 5′/3′ Kit (Clontech, Mountain View, CA, USA). For the 3′-RACE reaction, a nested universal primer (NUP) and a degenerate primer were designed based on the highly conserved region of the 5′-untranslated region from the Caerin family [18]. The degenerate primer sequence was 5′-GVCCTTGTAAAGACCAAVCATGGCTT-3′ [19]. The RACE products were purified using the E.Z.N.A.^®^ Cycle Pure Kit (Omega Bio-Tek, Norcross, GA, USA) and cloned into the pGEM^®^-T Easy Vector System (Promega, Madison, WI, USA). Finally, sequencing was performed by Eurofins Biosearch (Belfast, UK) as a commercial service.

### 2.3. Peptide Synthesis, Purification and Identification

Caerin 1.1-LC and its analogues were synthesised via solid-phase peptide synthesis (SPPS) using a Tribute 2-channel peptide synthesiser (Protein Technologies, Tucson, AZ, USA). The FITC-labelled peptide was synthesised by introducing 6-Ahx at the N-terminus of the peptide chain, which served as a spacer to minimise the influence of the FITC group on the peptide’s biological activity. The resin was shaken overnight in DMF with FITC (0.4 mmol, Fluorochem, Hadfield, UK) and DIPEA (0.3 mmol). Subsequently, the resin was washed three times with DMF, and the peptide was then cleaved to obtain the FITC-labelled sample under dark conditions. D-Leucine (D-Leu) and D-Valine (D-Val) residues were introduced at specific positions (position 16 or 17) to examine the influence of chirality on peptide conformation and activity. The crude peptides were purified by reversed-phase high-performance liquid chromatography (RP-HPLC) on a C18 column (250 × 21.2 mm, 5 μm, 300 Å; Phenomenex, Cheshire, UK) using an Agilent 1260 Infinity II system (Agilent, Cheshire, UK). A linear acetonitrile gradient elution method was employed for purification. The purified peptides were subsequently characterised by matrix-assisted laser desorption/ionisation time-of-flight mass spectrometry (MALDI-TOF MS) using a 4800 MALDI TOF/TOF instrument (Applied Biosystems, Foster City, CA, USA).

### 2.4. Antimicrobial Assay

The antimicrobial activities of the peptides were evaluated by determining their minimal inhibitory concentration (MIC) and minimal bactericidal concentration (MBC) values following reported protocols [20]. The tested microorganisms included seven Gram-negative bacterial strains (*Escherichia coli* ATCC 8739, *E. coli* ATCC-BAA 2340, *E. coli* NCTC 13846, *Klebsiella pneumoniae* ATCC 43816, *K. pneumoniae* ATCC BAA-2342, *Pseudomonas aeruginosa* ATCC 9027, *Acinetobacter baumannii* ATCC BAA-747), three Gram-positive bacterial strains (*Staphylococcus aureus* ATCC 6538, *Enterococcus faecalis* NCTC 12697, MRSA NCTC 12493, and the fungal strain *Candida albicans* ATCC 10231. For the assay, each bacterial strain was cultured overnight at 37 °C in 100 mL of medium in a flask. The cultures were then subcultured to reach the logarithmic growth phase and diluted to 5 × 10^5^ CFU/mL. Peptide samples were serially diluted two-fold in microplates, with concentrations ranging from 128 μM to 1 μM. The diluted bacterial suspensions were mixed with the peptide solutions and incubated overnight at 37 °C. The MIC values were determined by measuring the absorbance of each well using a Synergy HT plate reader (Agilent, Cheshire, UK). For MBC determination, aliquots from wells showing no visible growth were inoculated onto agar plates to assess bacterial viability. Each experiment was repeated independently three times and conducted in three independent replicates.

### 2.5. Time-Killing Assay

The time-killing assay was used to detect the killing kinetics of the peptide [21]. Overnight bacterial cultures were subcultured and adjusted to a concentration of 5 × 10^5^ CFU/mL in fresh medium. Bacterial suspensions were then exposed to peptide solutions at concentrations of 1×, 2×, and 4× MIC in 96-well plates. At designated time points, 10 μL aliquots from each treatment were mixed with 90 μL of sterile PBS (1:10 dilution) and spread onto agar plates for viable colony counting. Colony-forming units (CFU) were enumerated after incubation to determine bactericidal activity at each time point and peptide concentration. Each experiment was performed three times, with three replicates per experiment.

### 2.6. Salt and Serum Sensitivity Assay

The peptide sensitivity was determined in different salt- and serum-containing environments [22,23]. Bacterial strains were prepared following the MIC detection protocol described previously. To evaluate peptide sensitivity under physiological conditions, various concentrations of salt ions (150 mM NaCl, 4.5 mM KCl, 2 mM CaCl_2_, 1 mM MgCl_2_, 8 µM ZnCl_2_, 6 µM NH_4_Cl, or 4 µM FeCl_3_) and serum (10% FBS) were prepared in McCartney bottles prior to the addition of bacterial suspensions. The subcultured bacterial suspensions were then introduced to these modified media conditions. For MIC determination, the treated bacterial suspensions were diluted and dispensed into 96-well plates containing peptide solutions at graded concentrations. This assay allowed for the detection of potential alterations in MIC values, serving as an indicator of peptide sensitivity under different physiological conditions. All experiments were independently repeated three times, each with three replicates.

### 2.7. Haemolysis Assay

The peptide cytotoxicity against mammalian erythrocytes was evaluated by haemolysis assay on horse red blood cells (E&O Laboratories Ltd., Falkirk, UK) [21]. Fresh horse blood was washed three times with PBS and resuspended to a final concentration of 4% (*v*/*v*) in PBS. The diluted blood suspension was then mixed with peptide solutions at varying concentrations and incubated for 2 h at 37 °C. Red blood cell suspensions treated with 0.1% Triton X-100 and PBS were used as controls. The Triton X-100-treated suspension was the positive control (for 100% haemolysis), while the PBS-treated suspension was the blank control. Following incubation, the mixtures were centrifuged at 1000× *g* for 10 min to separate erythrocytes from the supernatant. The absorbance of the supernatants was detected at 570 nm. The percentage haemolysis was calculated by following the equation

Percentage Haemolysis = (Abs_Sample_ − Abs_Blank_)/(Abs_Positive_ − Abs_Blank_) × 100%

### 2.8. MTT Assay

The peptide’s cytotoxicity against normal cell lines was detected by the MTT assay [21]. Following resuscitation from cryopreservation (−80 °C), the cell line underwent multiple passages to ensure viability and adaptation. Cells were then seeded into 96-well plates and maintained at 37 °C in a 5% CO_2_ humidified atmosphere for 24 h to achieve proper adherence. After incubation, cells were serum-starved for 4 h using foetal bovine serum (FBS)-free medium (ThermoFisher, Waltham, MA, USA) to synchronise cell cycle progression. Peptide solutions were prepared at concentrations ranging from 128 μM to 1 μM (serial two-fold dilutions) and applied to the cells. Following peptide treatment, 10 μL of MTT (3-(4,5-dimethylthiazol-2-yl)-2,5-diphenyltetrazolium bromide) solution (5 mg/mL in PBS) was added to each well and incubated for 2 h at 37 °C. The medium was then carefully aspirated, and 100 μL of DMSO was added to dissolve the formazan crystals. Absorbance was measured at 570 nm using a microplate reader (Agilent, Cheshire, UK), with a reference wavelength of 630 nm to account for background interference. Experiments were conducted in triplicate and repeated three independent times.

### 2.9. In Vivo Toxicity and Antimicrobial Activity

Healthy *Galleria mellonella* (*G. mellonella*) larvae (Livefood UK Ltd., Somerset, UK) weighing 250 ± 25 mg were selected for each experimental group (n = 10 per group) [21]. The drug-resistant *E. coli* (ATCC BAA-2340) strain was cultured to the logarithmic phase and adjusted to 1 × 10^8^ CFU/mL in sterile PBS. Each larva was infected via injection of 10 μL bacterial suspension into the last proleg using a sterile syringe (1 h infection period). After infection, larvae received 10 μL peptide solutions at final concentrations of 1×, 2×, and 4× MIC (calculated based on average larval weight). A negative control group received 10 μL PBS. Injections were performed in the contralateral proleg using a microinjector (Hamilton, Birmingham, UK). Larvae were maintained at 37 °C in sterile Petri dishes with nutrition-free agar to prevent desiccation. Survival rates and phenotypic responses (melanisation, motility, pupation) were recorded every 24 h for five days. Larvae were considered dead upon absence of movement in response to tactile stimulation.

### 2.10. LPS Neutralisation Assay

Murine macrophage RAW 264.7 cells were cultured in 6-well plates at a density of 5 × 10^5^ cells per well and allowed to adhere for 24 h at 37 °C in a 5% CO_2_ humidified incubator. Cells were then stimulated with 100 ng/mL LPS in the presence or absence of test peptides at varying concentrations (typically 1–100 μM). Hydrocortisone (10 μg/mL) served as the positive control for anti-inflammatory activity. After 24 h of incubation, cell culture supernatants were collected and centrifuged at 300× *g* for 10 min to remove cellular debris. Tumour necrosis factor-alpha (TNF-α) secretion was quantified using a mouse TNF-α ELISA Development Kit (ThermoFisher Scientific, Waltham, MA, USA) according to the manufacturer’s protocol.

### 2.11. LPS-Binding Assay

The LPS-binding affinity of peptides was evaluated using a BODIPY-TR cadaverine (BC) displacement assay [24]. BC dye (ThermoFisher, Waltham, MA, USA) and LPS (*E. coli* O26:B6, Sigma-Aldrich, Dorset, UK) were diluted in Tris-HCl buffer to final concentrations of 5 μg/mL and 50 μg/mL, respectively, and incubated together for 4 h in the dark to form BC-LPS complexes. Peptide samples were then added to the mixture at final concentrations ranging from 1 to 128 μM (2-fold serial dilutions) in a black 96-well plate. After incubation, fluorescence was measured using a microplate reader with excitation at 590 nm and emission at 645 nm to quantify the displacement of BC from LPS by the peptides. Colistin (Sigma-Aldrich, Dorset, UK) was used as a positive control. Each experiment was carried out in three replicates and repeated on three separate occasions.

### 2.12. NPN Outer Membrane Permeability Assay

The outer membrane permeability of Gram-negative bacteria was evaluated using the 1-N-phenylnaphthylamine (NPN) uptake assay following reported methods [25]. Overnight bacterial cultures in LB medium were subcultured (200 μL inoculum in 25 mL fresh LB) for 2.5 h to the mid-log phase, then washed three times with 5 mM HEPES buffer (pH 7.2 containing 5 mM glucose) via centrifugation (3000× *g*) and adjusted to 1 × 10^7^ CFU/mL. Bacterial suspensions were mixed with NPN (final 10 μM) and test peptides at 1×, 2×, and 4× MICs, with fluorescence monitored dynamically for 2 h (excitation 350 nm/emission 420 nm) using a microplate reader. Melittin (10 μg/mL) and 5 mM HEPES buffer served as positive (100% permeability) and negative (0% permeability) controls, respectively. All experiments were performed in triplicate.

### 2.13. ONPG Inner Membrane Permeability Assay

The peptide influence on *E. coli* inner membrane permeability was evaluated by the ONPG assay [19,26]. The overnight-incubated bacterial suspension was subcultured in 25 mL LB medium with 2% lactose until logarithmic phase. The bacterial suspension (*E. coli* ATCC BAA-2340) was washed with PBS three times through centrifuging at 3000× *g*. Then it was resuspended to a density of OD_600_ = 0.5. The resuspended bacterial solution was diluted ten-fold by PBS buffer with 1.5 mM ONPG. The diluted bacterial solution was mixed with the sample peptide at 1× MIC, 2× MIC and 4× MIC for 2 h, and dynamically monitored in a plate reader at a 460 nm wavelength to detect the absorbance alteration. Melittin (Sigma-Aldrich, Dorset, UK) was selected as a positive control and PBS solution was served as a negative control. The experiments were replicated three times.

### 2.14. Bacterial Membrane Permeability Assay

The effect of peptides on bacterial membrane permeability was assessed using SYTOX Green nucleic acid stain (ThermoFisher, Waltham, MA, USA) as previously described [27]. *E. coli* ATCC BAA-2340 were grown in 25 mL of tryptic soy broth (TSB) for 2.5 h to the mid-log phase, then washed three times with a solution containing 5% TSB in 0.85% NaCl by centrifugation (3000× *g*, 10 min). The bacterial suspension was adjusted to an optical density of 0.7 at 590 nm (OD_590_) in the same buffer. In a black 96-well plate, the bacterial suspension was combined with peptide solutions (1×, 2×, and 4× MIC) and SYTOX Green (final concentration 1 μM). Fluorescence intensity (excitation 485 nm/emission 520 nm) was monitored kinetically for 2 h using a microplate reader. Membrane permeability was quantified by relative fluorescence units, with 0.1% Triton X-100-treated cells as a positive control (100% permeability) and untreated cells as a negative control (0% permeability). All experiments were performed in triplicate.

### 2.15. Localisation of FITC-Labelled Peptide on Bacteria

Peptide action location was visualised according to previous protocols [28,29,30]. NucBlue™ Live ReadyProbes™ Reagent (Hoechst 33342, ThermoFisher, Waltham, MA, USA) and FM™ 4-64 Dye (ThermoFisher, Waltham, MA, USA) were used as nuclear staining and membrane staining dyes. Briefly, the bacteria were incubated to the logarithmic phase and incubated with FITC-labelled peptide for 2 h. After incubation, bacteria were stained with Hoechst 33,342 and FM 4-64 for 20 min. After washing with PBS to remove the unbound dye, 5 μL of bacterial suspension was loaded on agar and observed by a fluorescent microscope (DMi8, Leica, Nussloch, Germany) with a 100× oil-immersion objective.

### 2.16. Membrane Depolarisation Assay

The peptide’s influence on membrane potential was detected according to previous studies [31]. The bacterial strains were cultured to the logarithmic phase in lysogeny broth (LB) medium and the bacterial suspension was washed with PBS following 3000× *g* centrifugation. The washed bacteria were diluted to OD_600_ = 0.05 with PBS. Disc_3_(5) was mixed with the suspension to a final concentration of 0.4 μM and incubated for 1 h at 37 °C. Then the incubated suspension was added with the peptide sample at 0.25× MIC, 0.5× MIC, 1× MIC, 2× MIC, and 4× MIC and monitored on a plate reader for 30 min with the excitation wavelength set at λ = 485 nm and the emission wavelength set at λ = 645 nm. Experiments were performed in triplicate and independently repeated three times.

### 2.17. Intracellular ATP Assay

Variations in intracellular ATP levels, with or without peptide treatment, were measured using an ATP assay kit (Abcam, Cambridge, UK) as previously described [21]. Briefly, log-phase bacteria were diluted and exposed to peptides at gradient concentrations for 1 h at 37 °C. Thereafter, the detergent provided in the kit was added to lyse the bacteria, and the lysates were subsequently mixed with the substrate solution.

### 2.18. Statistical Analysis

All data were analysed using GraphPad Prism 10 (GraphPad, San Diego, CA, USA). Values are presented as mean ± standard deviation (SD), with error bars representing SD. Statistical significance is indicated as ns (*p* ≥ 0.05) and **** *p* < 0.0001.

## 3. Results

### 3.1. Molecular Cloning of Caerin 1.1-LC Precursor-Encoding cDNA

**Figure 1:**
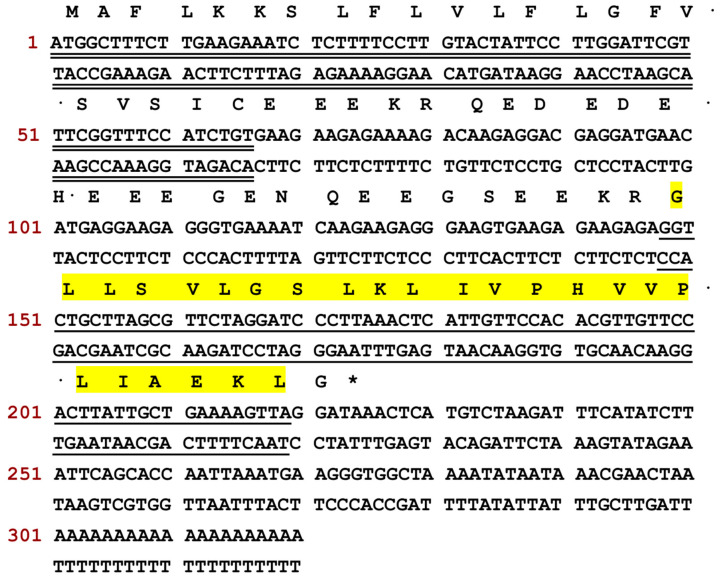
The nucleotide sequence and translated ORF amino acids of a cDNA encoding a novel peptide from *L. caerulea* skin secretions. The nucleotide sequence of the signal peptide is double-underlined. The nucleotide sequence of mature peptide Caerin 1.1-LC is single-underlined and marked in yellow. The stop codon is indicated by an asterisk.

**Figure 2:**
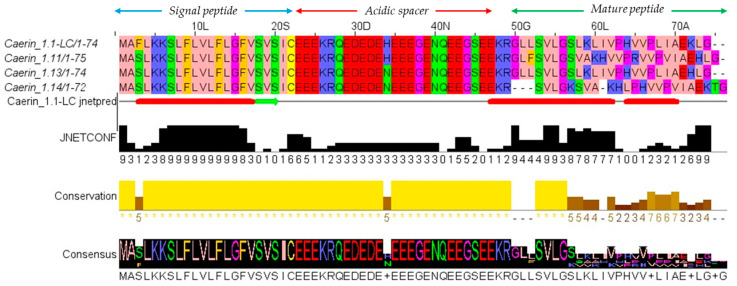
Multiple sequence alignment results of Caerin 1.1-LC with similar peptides found in NCBI-BLAST database. The Zappo colour scheme is used to label and reveal patterns of variations. JNetPred depicts the results of the secondary structure prediction. The α-helical structures are represented by red tubes, and the β-sheet structures are indicated with green arrows. JNetCONF indicates the confidence level of the prediction, where a higher value corresponds to greater confidence. The symbol below the conservation section is an asterisk, indicating identical amino acids.

A cDNA encoding a short peptide was identified through molecular cloning of the skin secretions of *Litoria caerulea*. The nucleotide sequence and its corresponding amino acid sequence are shown in Figure 1. The deduced open reading frame (ORF) of the cDNA consisted of 320 base pairs encoding 74 amino acids, comprising a 22-residue N-terminal putative signal peptide, an acidic residue-rich spacer domain, a 24-residue mature peptide, and a C-terminal processing and amidation site. The precursor peptide sequence was submitted to the NCBI BLAST tool (https://blast.ncbi.nlm.nih.gov/Blast.cgi, accessed on 4 November 2025) for further analysis. Sequence alignment results, presented in Figure 2, indicated that this peptide represents a novel AMP and shares a high degree of identity (88–96%) with three members of the Caerin family, based on the alignment of the full precursor amino acid sequences. The alignment was visualised using Jalview. Accordingly, this novel peptide was named Caerin 1.1-LC, classifying it within the Caerin 1.1 family. The nucleotide sequence of Caerin 1.1-LC has been deposited in GenBank under the accession number PX310544 (accession on 22 October 2025).

### 3.2. Peptide Design, Characterisation and Structure Analysis

To investigate the structure–activity relationship of Caerin 1.1-LC, we designed a series of peptide analogues with targeted modifications of cationicity, hydrophobicity, and hinge structure. To enhance electrostatic interactions with the negatively charged bacterial membranes, basic amino acids were introduced at different positions to optimise the net positive charge and charge distribution along the peptide chain (Caerin 1.1-LC, Caerin 1.1-LC 11K, Caerin 1.1-LC 11K,22K, Caerin 1.1-LC 7.11.19K) (Table 1). To assess the impact of hydrophobicity, selected residues were substituted with more hydrophobic amino acids (Caerin 1.1-LC 11K.12K.12W, Caerin 1.1-LC 11K.12K.19W) (Table 2).

In parallel, the functional role of the hinge structure was systematically explored. First, the hinge structure was broken to generate analogue Caerin 1.1-LC 2P-2A to evaluate its impact on antimicrobial activity. Subsequently, further hinge-targeted modifications were introduced to modulate flexibility (Caerin 1.1-LC PGGGP), hydrophobicity (Caerin 1.1-LC PHLLP, Caerin 1.1-LC PHIIP, Caerin 1.1-LC PHWWP), and net charge (Caerin 1.1-LC PRVVP, Caerin 1.1-LC PKVVP). Based on bioactivity screening, Caerin 1.1-LC 7.11.19K, which exhibited the highest selectivity, was selected as a template for further hinge optimisation. Three derivatives were subsequently generated by reducing hinge hydrophobicity or incorporating D-amino acids (Caerin 1.1-LC 11K.19K.PKAVP, Caerin 1.1-LC PKAL(D)P, Caerin 1.1-LC 7.11.19K.V(D)V(D)). Caerin 1.1-LC and its analogues were synthesised via SPPS, characterised by MALDI-TOF MS, and purified using RP-HPLC. All peptides used in subsequent assays had a purity exceeding 95% (Figure S1).

### 3.3. Antimicrobial Activity

The antimicrobial activities of Caerin 1.1-LC and its analogues are summarised in Table 3. The parent peptide displayed broad-spectrum but relatively mild activity, showing stronger effects against Gram-negative bacteria compared with the tested Gram-positive strains. Structural modifications enhanced activity in various ways. Increasing net charge and hydrophobicity on the helical motif (e.g., Caerin 1.1-LC 11K, 11K.22K, 11K.22K.12W, 11K.22K.19W, 7.11.19K) generally improved potency, with Caerin 1.1-LC 11K.22K.12W showing the strongest effect (MIC 1–32 µM). Alterations in the hinge motif had more variable effects. Reduced helicity, as seen in Caerin 1.1-LC PHWWP and PGGGP, resulted in poor activity, whereas replacing proline with alanine (2P-2A) to increase helicity completely abolished activity. Conversely, modifications that increased net charge or hydrophobicity in the hinge motif (PHIIP, PHLLP, PRVVP, PKVVP) led to dramatic improvements. Substituting D-form amino acids in the hinge region (11K.19K.PKAL(D)P, 7.11.19K.V(D)V(D)) caused only slight changes in antimicrobial activity, particularly against Gram-negative bacteria, compared to the highly active Caerin 1.1-LC 7.11.19K.

### 3.4. Haemolysis, Therapeutic Index and Cytotoxicity

The original peptide, Caerin 1.1-LC, exhibited mild haemolytic activity with an HC10 value of 37.5 μM, resulting in low therapeutic indices (TIs) of 0.47 and 2.63 against Gram-negative and Gram-positive bacteria, respectively (Table 4). Increasing hydrophobicity in the helix and hinge motifs of peptide analogues (Caerin 1.1-LC 11K.12K.12W, Caerin 1.1-LC 11K.12K.19W, Caerin 1.1-LC PHLLP, Caerin 1.1-LC PHIIP, Caerin 1.1-LC PKAL(D)P) led to a significant increase in haemolytic activity and reduced TIs, indicating low selectivity. Analogues modified by introducing positively charged residues (Caerin 1.1-LC PRVVP, Caerin 1.1-LC PKVVP) also showed increased amphipathicity in the helix motif, enhancing haemolytic activity with HC10 values of 25.8 μM and 21.2 μM, respectively. However, these modifications did not notably affect the TIs. Caerin 1.1-LC 7.11.19K, which exhibited the lowest haemolytic activity among the helix-modified peptides, was selected for further optimisation. The haemolytic activity was successfully reduced by decreasing hydrophobicity or substituting hydrophobic amino acids in the hinge region with D-amino acids. Caerin 1.1-LC 11K.19K.PKAVP and Caerin 1.1-LC 7.11.19K.V(D)V(D) showed the lowest cytotoxicity, with Caerin 1.1-LC 7.11.19K.V(D)V(D) exhibiting the best TI of 26.3 against Gram-negative bacteria. Based on these results, Caerin 1.1-LC 7.11.19K.V(D)V(D) and the original peptide, Caerin 1.1-LC, were selected for further research to elucidate their mechanism of action and assess their therapeutic potential against drug-resistant *E. coli* (ATCC BAA-2340).

Human embryonic lung fibroblasts (MRC-5), human embryonic kidney cells (HEK 293), human microvascular endothelial cells (HMEC-1), human keratinocytes (HaCaT), and murine macrophages (RAW 264.7) were used to evaluate the cytotoxicity of Caerin 1.1-LC and its analogue Caerin 1.1-LC 7.11.19K.V(D)V(D). As shown in Table 5, the parent peptide exhibited varying degrees of cytotoxicity across the tested cell lines. Among them, RAW 264.7 cells were the most susceptible strain, displaying the lowest IC50 value of 6.0 μM. In contrast, Caerin 1.1-LC 7.11.19K.V(D)V(D) demonstrated only weak cytotoxicity, with the highest IC50 value of 129.6 μM observed in HEK 293 cells, substantially higher than that of the parent peptide.

### 3.5. Salt Sensitivity of Caerin 1.1-LC and Its Analogues

The sensitivity of the peptides’ antimicrobial activity under different environmental conditions was assessed by measuring changes in MIC values. *E. coli* (ATCC-BAA 2340), the most susceptible strain, was used. As shown in Table 6, K^+^, NH_4_^+^, and Fe^3+^ had no notable effect on the antimicrobial activity of the peptides. NaCl increased the MIC of Caerin 1.1-LC 7.11.19K.V(D)V(D) by four-fold, while other peptides remained unaffected. In contrast, Mg^2+^, Ca^2+^, and FBS varying degrees of inhibition, increasing MIC values by 2- to 64-fold for tested peptides.

### 3.6. In Vivo Antimicrobial Activity on Larvae Model

**Figure 3:**
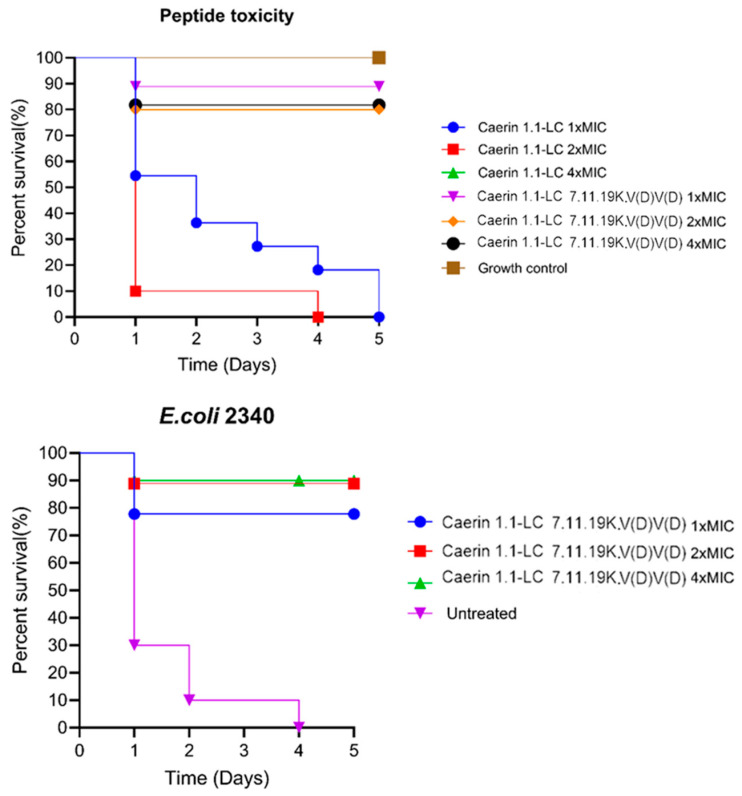
Peptide toxicity and in vivo antibacterial activity in *E. coli*-infected waxworms. For toxicity testing, both peptides were evaluated at concentrations of 1× MIC, 2× MIC, and 4× MIC. The green line (Caerin 1.1-LC 4× MIC) completely overlaps with the red line (Caerin 1.1-LC 2× MIC). An equivalent injection volume of PBS served as the growth control group. For the antibacterial study, an equal volume of bacterial suspension without peptide treatment was used as the untreated control group.

To evaluate the in vivo antimicrobial activity of the peptides, cytotoxicity was first assessed by applying different concentrations to larval worms. As shown in Figure 3, Caerin 1.1-LC exhibited high cytotoxicity compared to Caerin 1.1-LC 7.11.19K.V(D)V(D), resulting in complete mortality by day four or five post-injection. Consequently, Caerin 1.1-LC 7.11.19K.V(D)V(D) was selected for subsequent treatment of *E. coli* infection in larval worms, including the highly drug-resistant strain *E. coli* (ATCC BAA-2340). Both tested concentrations of Caerin 1.1-LC 7.11.19K.V(D)V(D) showed substantial therapeutic effects. After five days of treatment, survival rates increased to 80% at 1× MIC and 90% at 2× MIC and 4× MIC.

### 3.7. LPS Neutralisation Activity

**Figure 4:**
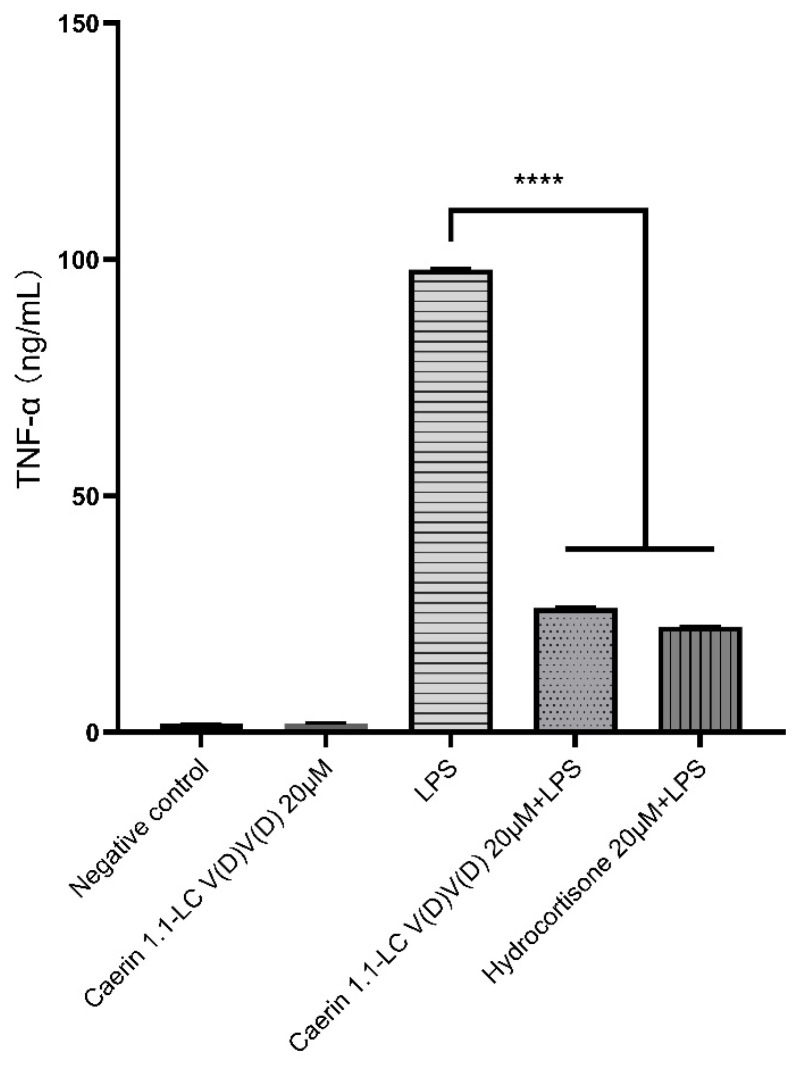
Peptide Caerin 1.1-LC 7.11.19K.V(D)V(D) endotoxin neutralisation activity. Murine macrophage RAW 264.7 was stimulated by LPS for TNF-α quantitative analysis. The statistical significance is indicated as **** (*p* < 0.0001) versus LPS group.

Peptide endotoxin-neutralisation activity was quantitatively assessed using an LPS-stimulated RAW 264.7 model (Figure 4). Caerin 1.1-LC 7.11.19K.V(D)V(D) alone did not induce noticeable TNF-α production in RAW 264.7 cells. LPS stimulation significantly increased the expression of the pro-inflammatory cytokine TNF-α in murine macrophages RAW 264.7 cells, reaching approximately 100 ng/mL. When administered after LPS stimulation, Caerin 1.1-LC 7.11.19K.V(D)V(D) exhibited strong endotoxin-neutralising activity, significantly reducing TNF-α levels to 26.3 ng/mL, which is comparable to the effect of hydrocortisone at the same concentration (approximately 22.3 ng/mL).

### 3.8. Time-Killing Kinetics Studies

**Figure 5:**
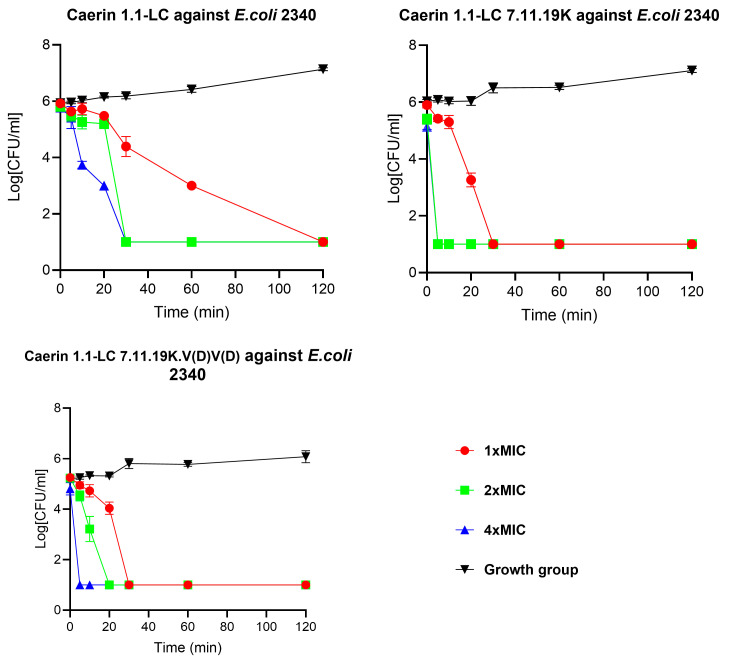
Time–kill curves of Caerin 1.1-LC, Caerin 1.1-LC 7.11.19K, and Caerin 1.1-LC 7.11.19K.V(D)V(D) against *E. coli* (ATCC BAA-2340). The test duration was 120 min, with peptide concentrations ranging from 1× MIC to 4× MIC. Bacteria without peptide treatment were used as the growth control.

Time–kill kinetics assays were performed to evaluate the bactericidal rates of the peptides. As shown in Figure 5, Caerin 1.1-LC exhibited rapid bactericidal activity, eradicating bacteria within 30 min at 2× and 4× MIC, while complete killing at 1× MIC required 120 min. Caerin 1.1-LC 7.11.19K, with enhanced cationicity, displayed an even faster killing rate, achieving complete eradication within 5 min at 2× and 4× MIC and within 30 min at 1× MIC. Caerin 1.1-LC 7.11.19K.V(D)V(D) showed a comparable killing profile, also eliminating bacteria within 30 min.

### 3.9. LPS-Binding Affinity

**Figure 6:**
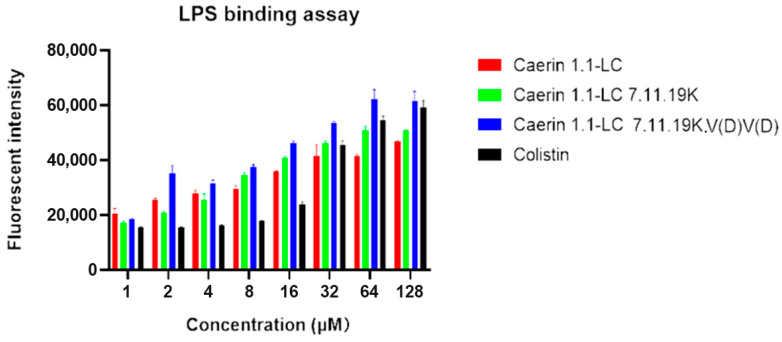
LPS-binding affinity of peptides Caerin 1.1-LC, Caerin 1.1-LC 7.11.19K and Caerin 1.1-LC 7.11.19K.V(D)V(D); same tested concentration of colistin was chosen as positive control. Data are shown as means ± standard deviations of three independent experiments.

The results of the peptide–LPS-binding assay are shown in Figure 6. The template peptide, Caerin 1.1-LC, exhibited relatively weaker binding affinity to LPS compared with the other tested analogues. Although the net charge disruption along the peptide chains was the same, Caerin 1.1-LC 7.11.19K.V(D)V(D) demonstrated better binding affinity than Caerin 1.1-LC 7.11.19K. Both of these peptides showed superior LPS-binding affinity compared with the positive control, Colistin.

### 3.10. Outer Membrane Permeabilisation

**Figure 7:**
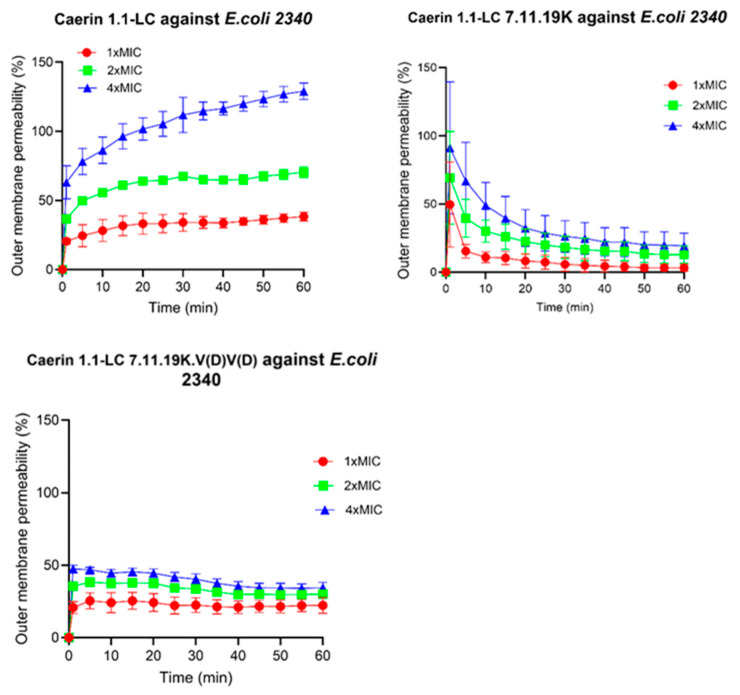
Effect of peptides’ influence on bacterial outer membrane permeability. The percentage of permeability was calculated by the ratio of sample fluorescence intensity to 4× MIC (16 μM) melittin treatment fluorescence intensity. The error bars represent the SD of six replications.

The effect of peptides on *E. coli* outer membrane permeability was assessed using the NPN uptake assay. As shown in Figure 7, the template peptide Caerin 1.1-LC induced a pronounced alteration in outer membrane permeability, with the highest effect observed at 4× MIC. For Caerin 1.1-LC 7.11.19K, although it initially caused a rapid and substantial increase in outer membrane permeability, the fluorescence intensity decreased sharply over time, leading to an apparent reduction in the calculated membrane permeabilisation rate. In contrast, Caerin 1.1-LC 7.11.19K.V(D)V(D) exhibited a much weaker effect, reaching only approximately 50% of the maximum permeability at the highest tested concentration.

### 3.11. Intracellular Membrane Permeability

**Figure 8:**
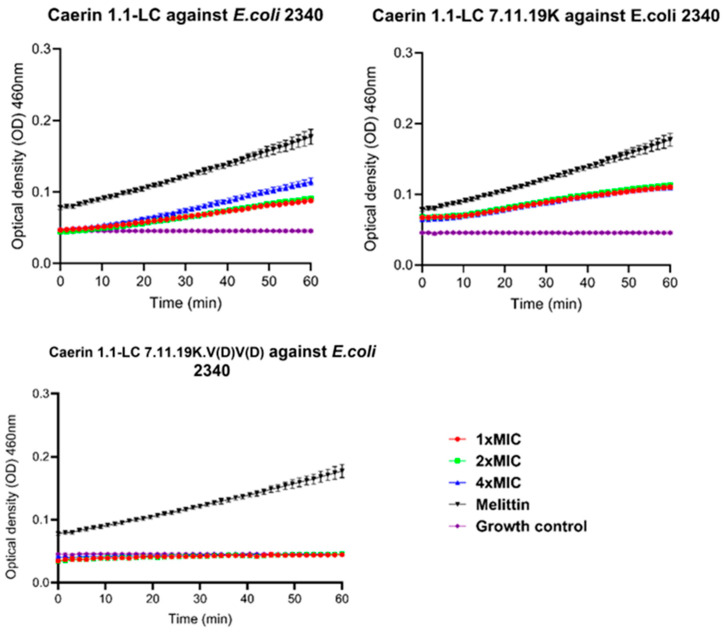
Peptide influence on *E. coli* (ATCC BAA-2340) intracellular membrane permeability. Same volume of PBS treatment was set as growth control. Melittin (4× MIC, 16 μM) treatment was set as positive control. Error bars represent SD of nine independent replications.

The effect of peptides on intracellular membrane permeability was evaluated using the ONPG assay. As shown in Figure 8, the original peptide Caerin 1.1-LC exhibits a concentration-dependent increase in intracellular membrane permeability, with both tested concentrations producing measurable changes. A similar trend was observed for Caerin 1.1-LC 7.11.19K, which also induced increased membrane permeability within the test period. In contrast, treatment with Caerin 1.1-LC 7.11.19K.V(D)V(D) did not produce any detectable changes in intracellular membrane permeability at any concentration tested against *E. coli* (ATCC BAA-2340).

### 3.12. Further Validation of Membrane Integrity

**Figure 9:**
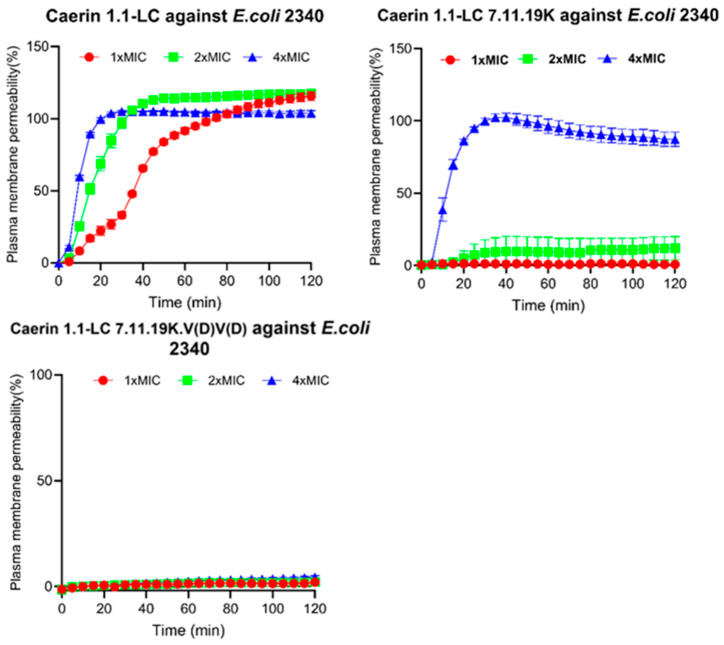
Effect of peptides on bacterial plasma membrane permeability. The percentage of membrane permeability was calculated as the ratio of sample fluorescence intensity to the fluorescence intensity induced by melittin treatment. Error bars represent the standard deviation (SD) of nine independent replicates.

The effect of Caerin 1.1-LC and its analogues on bacterial membrane integrity was evaluated using SYTOX Green dye (Figure 9). The parent peptide Caerin 1.1-LC induced rapid and strong membrane permeabilisation, indicating that its bactericidal activity is primarily mediated through disruption of membrane integrity. In contrast, the peptide Caerin 1.1-LC 7,9,11K, which carries increased cationicity, showed low membrane permeabilisation at 1× and 2× MIC, but a clear concentration-dependent effect was observed as the concentration increased, with a pronounced increase from 2× MIC to 4× MIC. Notably, Caerin 1.1-LC 7.11.19K.V(D)V(D) did not induce detectable membrane permeabilisation across the tested concentration range, suggesting that it may exert antibacterial activity via a mechanism distinct from direct membrane disruption.

### 3.13. Localisation of FITC-Labelled Peptides

**Figure 10:**
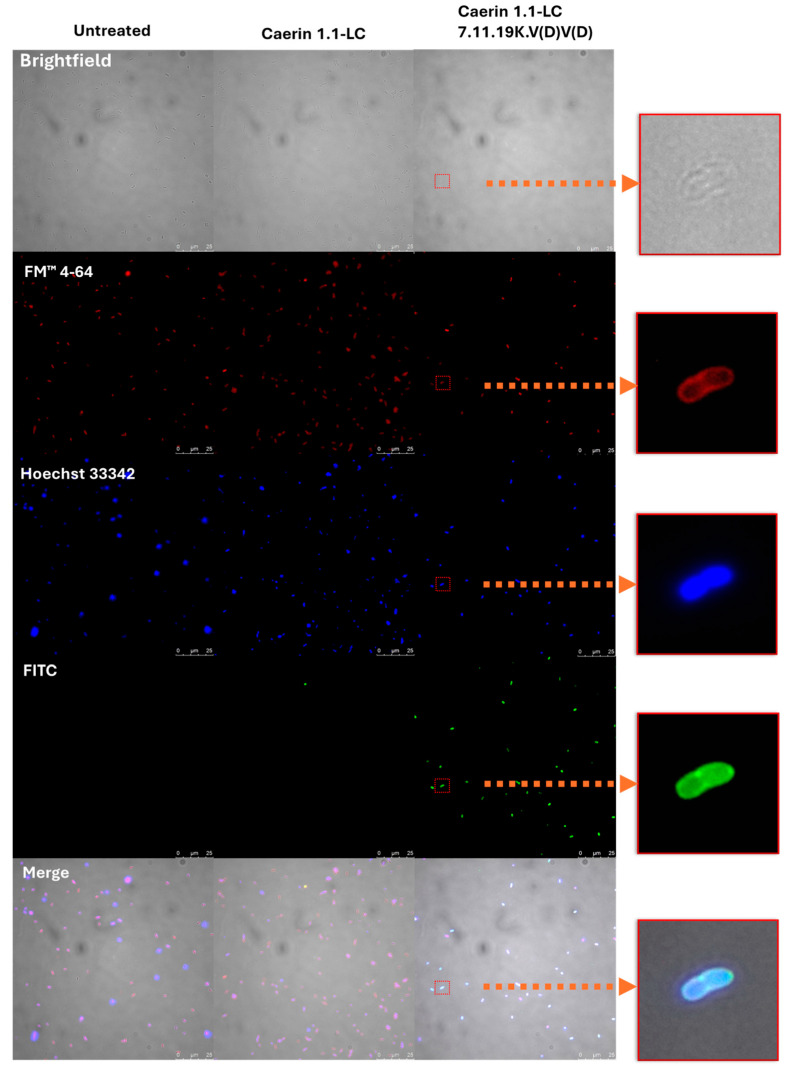
Fluorescence images of Caerin 1.1-LC 7.11.19K.V(D)V(D)-treated *E. coli* (ATCC BAA-2340). FM 4-64 (red) labels the plasma membrane, HOECHST 33,342 (blue) stains the cell nucleus, and FITC (green) is conjugated to our peptides to track their localisation. The scale bar is 25 µm.

To further investigate the mechanism of action of the peptides, both the parent peptide Caerin 1.1-LC and the V(D)V(D) analogue were labelled with FITC and incubated with *E. coli* (ATCC BAA-2340) cells. As shown in Figure 10, Caerin 1.1-LC did not exhibit detectable intracellular fluorescence. In contrast, FITC-labelled Caerin 1.1-LC 7.11.19K.V(D)V(D) displayed strong fluorescence inside bacterial cells, indicating membrane penetration and intracellular localisation.

### 3.14. Membrane Depolarisation Activity

**Figure 11:**
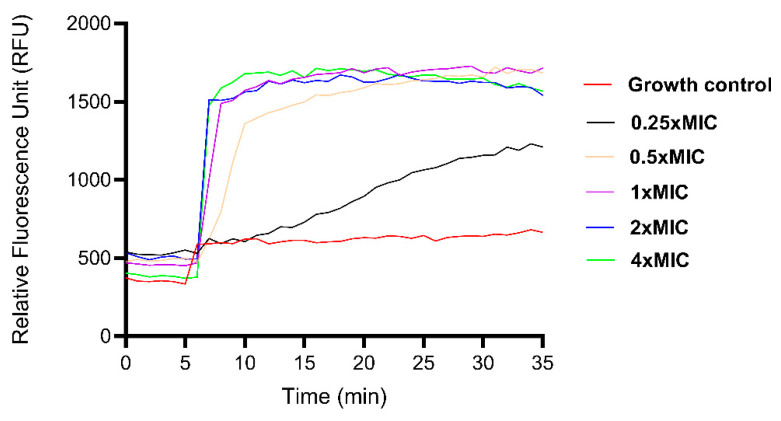
Effect of Caerin 1.1-LC 7.11.19K.V(D)V(D) on cytoplasmic membrane depolarisation against *E. coli* (ATCC BAA-2340).

The effect of Caerin 1.1-LC 7.11.19K.V(D)V(D) on bacterial membrane potential was evaluated using the membrane-sensitive dye DiSC_3_(5). As shown in Figure 11, treatment with the peptide resulted in a rapid and concentration-dependent increase in fluorescence intensity, indicating immediate depolarisation of the bacterial membrane. This effect was observed at all tested concentrations except 0.25× MIC, where great depolarisation was not detected until 5 min after peptide addition. These findings suggest that Caerin 1.1-LC 7.11.19K.V(D)V(D) disrupts membrane potential efficiently, contributing to its antibacterial activity.

### 3.15. Disruption of the Intracellular ATP Level

**Figure 12:**
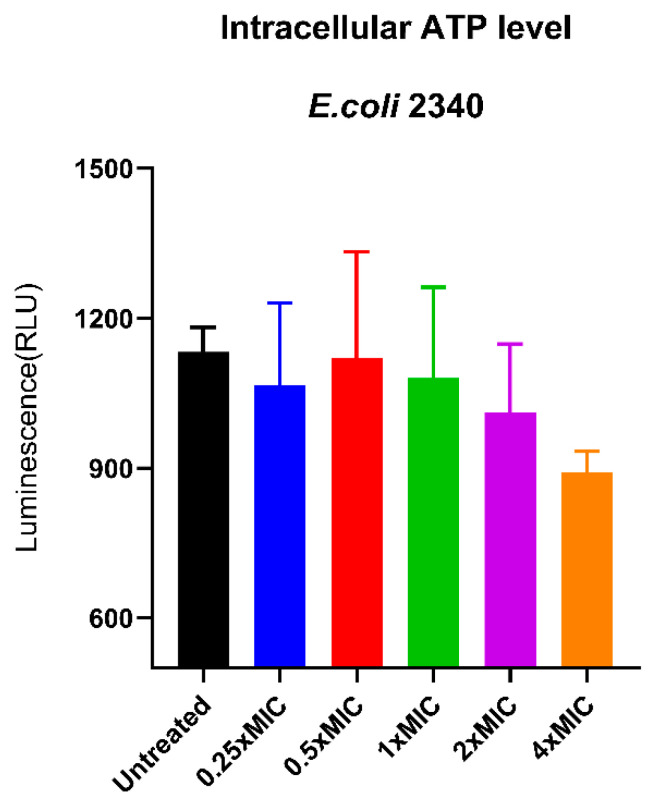
Intracellular ATP level in *E. coli* (ATCC BAA-2340) with/without the treatment of Caerin 1.1-LC 7.11.19K.V(D)V(D).

Proton motive force (PMF) disruption is often associated with perturbations in bacterial energy metabolism. To investigate whether the Caerin 1.1-LC 7.11.19K.V(D)V(D) peptide affects bacterial ATP production, the intracellular ATP levels of peptide-treated bacteria were assessed. As shown in Figure 12, the presence of the peptide remarkably impacted bacterial ATP levels. Especially, as the peptide concentration increased from 1× MIC to 4× MIC, a notable decrease in intracellular ATP was observed, indicating that Caerin 1.1-LC 7.11.19K.V(D)V(D) impairs cellular energy homeostasis in a concentration-dependent manner.

## 4. Discussion

In this study, we systematically investigated the structural determinants of Caerin 1.1-LC antimicrobial activity, focusing on both the helix and hinge motifs. The parent peptide exhibited broad-spectrum antimicrobial activity but poor selectivity due to strong haemolysis. Through rational design, we modulated net charge, hydrophobicity, and hinge flexibility to enhance selectivity while retaining antimicrobial potency. The Caerin family is characterised by a helix–hinge–helix motif and eliminates bacteria through a carpet-like membrane disruption model [32]. Hydrophobicity, charge, and helicity are key factors influencing biological activity and selectivity. Modifications in the helix region, including increased cationicity or hydrophobicity (e.g., Caerin 1.1-LC 11K, 11K.22K.12W, 7.11.19K), generally enhanced antimicrobial activity, consistent with the established role of electrostatic and hydrophobic interactions in membrane targeting. However, excessive hydrophobicity was associated with increased cytotoxicity, highlighting the delicate balance between potency and selectivity.

The hinge motif, primarily composed of proline residues, emerged as a critical determinant of activity, facilitating the peptide membrane disruption mechanism and subsequent antimicrobial efficacy [11]. Previous studies have reported that proline residues act as helix breakers, introducing a bend in the helix that optimises membrane binding and maximises membrane disruption [12]. In this study, complete replacement of proline residues with alanine (Caerin 1.1-LC 2P-2A) abolished antimicrobial activity, confirming that prolines are essential for maintaining the functional bend required for optimal membrane interaction. Alterations in residues between hinge prolines (e.g., Caerin 1.1-LC PGGGP, PHWWP) revealed that either increased flexibility or rigid hydrophobic residues can negatively impact helicity and antimicrobial efficacy, demonstrating that both hinge rigidity and local secondary structure are finely tuned for activity [33,34]. Notably, the introduction of D-amino acids in the hinge motif (Caerin 1.1-LC 7.11.19K.V(D)V(D)) markedly reduced cytotoxicity while maintaining strong activity against Gram-negative bacteria, resulting in a more than 50-fold increase in the therapeutic index. Previous studies have shown that incorporation of non-natural D-amino acids at functional residues can enhance selectivity, likely by decreasing peptide affinity for mammalian cells and thus minimising cytotoxicity [35,36]. The reduced cytotoxicity contributed substantially to the highest TI values, particularly against Gram-negative bacteria. Consequently, Caerin 1.1-LC 7.11.19K.V(D)V(D) was selected for further investigation to explore additional functional properties and evaluate its potential as a therapeutic agent.

Salt ion and serum influence experiments demonstrated that the antibacterial activity of the tested peptides was primarily attenuated by calcium, magnesium, sodium ions, and FBS. Negatively charged components on Gram-negative bacterial membranes, such as LPS, contain divalent cation binding sites that can be competitively occupied by calcium and magnesium ions, reducing peptide–membrane interactions [37]. High-NaCl conditions can also induce conformational changes in LPS, further diminishing AMP binding affinity [38]. Beyond direct antimicrobial effects, some AMPs are known to neutralise endotoxin, thereby mitigating inflammation [39,40]. Consistently, Caerin 1.1-LC 7.11.19K.V(D)V(D) exhibited comparable inhibition of pro-inflammatory cytokine production to the conventional anti-inflammatory drug hydrocortisone, highlighting its potential as a novel anti-infective and anti-inflammatory therapeutic agent.

Mechanistic studies suggest that the D-isomer, Caerin 1.1-LC 7.11.19K.V(D)V(D), functions primarily through a non-lytic pathway, binding outer membrane LPS and translocating intracellularly rather than inducing substantial cytoplasmic membrane permeabilization or severe membrane damage. During this process, the peptide causes membrane depolarization, disrupting the PMF and impairing ATP production, ultimately leading to bacterial cell death. This mechanism explains why standard dye leakage assays failed to detect membrane disruption despite potent antibacterial activity. Recent studies on AMPs, such as Piscidin 1, indicate that small or transient pores can facilitate peptide translocation and local membrane perturbation without measurable dye leakage, consistent with our observations [41].

Modification studies further highlight the critical role of the hinge motif in the antibacterial mode of action. The introduction of D-valine residues within the hinge may alter the local twist and orientation of the peptide, promoting efficient interaction with the membrane and facilitating intracellular translocation. While membrane depolarisation and energy disruption appear to be the primary mechanisms, the peptide may also engage intracellular targets, which cannot be excluded as contributing factors to its antimicrobial efficacy. Overall, this alternative, non-lytic mechanism provides a promising strategy to enhance selectivity while maintaining strong antimicrobial potency, demonstrating the potential of hinge-targeted and D-amino-acid-based modifications for rational AMP design. Moreover, the ability of Caerin 1.1-LC 7.11.19K.V(D)V(D) to translocate across membranes, reminiscent of CPPs, suggests additional applications: it could serve as a carrier to deliver other antimicrobial agents or, when combined with functional motifs, be engineered into novel STAMPs for precise and potent bacterial targeting [42].

## 5. Conclusions

In this work, modifications of a novel Caerin 1 peptide, Caerin 1.1-LC, revealed the critical role of the hinge structure in Caerin 1-like peptides, particularly through the introduction of D-amino acids into the hinge region of one analogue. Beyond providing insights for AMP design, Caerin 1.1-LC 7.11.19K.V(D)V(D) exhibited remarkable selectivity against Gram-negative bacteria, efficiently eliminating drug-resistant *E. coli* within 30 min while minimising the pro-inflammatory effects of LPS. Notably, the non-lytic mechanism of the Caerin 1.1-LC 7.11.19K.V(D)V(D) allows it to first bind LPS, penetrate the bacterial membrane, and translocate intracellularly, during which it disrupts cellular energy via membrane depolarisation. This CPP-like property also suggests potential applications as a novel cargo for conjugation with other antimicrobial agents, enabling precise and efficient therapeutic interventions.

## Figures and Tables

**Table 1:** Mature peptide sequences of Caerin 1.1-LC analogues.

Peptide Name	Peptide Sequence
Caerin 1.1-LC	GLLSVLGSLKLIVPHVVPLIAEKL-NH_2_
Caerin 1.1-LC-11K	GLLSVLGSLK**K**IVPHVVPLIAEKL-NH_2_
Caerin 1.1-LC-11K.22K	GLLSVLGSLK**K**IVPHVVPLIA**K**KL-NH_2_
Caerin 1.1-LC-11K.22K.12W	GLLSVLGSLK**KW**VPHVVPLIA**K**KL-NH_2_
Caerin 1.1-LC-11K.22K.19W	GLLSVLGSLK**K**IVPHVVP**W**IA**K**KL-NH_2_
Caerin 1.1-LC 7.11.19K	GLLSVL**K**SLK**K**IVPHVVP**K**IAEKL-NH_2_
Caerin 1.1-LC 2P-2A	GLLSVLGSLKLIV**A**HVV**A**LIAEKL-NH_2_
Caerin 1.1-LC PGGGP	GLLSVLGSLKLIVP**GGG**PLIAEKL-NH_2_
Caerin 1.1-LC PRVVP	GLLSVLGSLKLIVP**R**VVPLIAEKL-NH_2_
Caerin 1.1-LC PKVVP	GLLSVLGSLKLIVP**K**VVPLIAEKL-NH_2_
Caerin 1.1-LC PHLLP	GLLSVLGSLKLIVPH**LL**PLIAEKL-NH_2_
Caerin 1.1-LC PHIIP	GLLSVLGSLKLIVPH**II**PLIAEKL-NH_2_
Caerin 1.1-LC PHWWP	GLLSVLGSLKLIVPH**WW**PLIAEKL-NH_2_
Caerin 1.1-LC 11K.19K.PKAVP	GLLSVLGSLK**K**IVP**KA**VP**K**IAEKL-NH_2_
Caerin 1.1-LC 7.11.19K.V(D)V(D)	GLLSVL**K**SLK**K**IVPH***vv***P**K**IAEKL-NH_2_
Caerin 1.1-LC PKAL(D)P	GLLSVLGSLKLIVP**KA*l***PKIAEKL-NH_2_

Boldface indicates the positions where L-amino acids were substituted. Lowercase and italics indicate the positions where D-amino acids were substituted.

**Table 2:** The hydrophobicity and net charge (pH 7) of peptides.

Peptide Name	Length (AA)	Hydrophobicity *	Net Charge at pH 7
Caerin 1.1-LC	24	0.815	+2
Caerin 1.1-LC-11K	24	0.703	+3
Caerin 1.1-LC-11K.22K	24	0.688	+5
Caerin 1.1-LC-11K.12K.12W	24	0.707	+5
Caerin 1.1-LC-11K.12K.19W	24	0.711	+5
Caerin 1.1-LC 7.11.19K	24	0.550	+5
Caerin 1.1-LC 2P-2A	24	0.781	+2
Caerin 1.1-LC PGGGP	24	0.708	+2
Caerin 1.1-LC PRVVP	24	0.768	+3
Caerin 1.1-LC PKVVP	24	0.768	+3
Caerin 1.1-LC PHLLP	24	0.855	+2
Caerin 1.1-LC PHIIP	24	0.863	+2
Caerin 1.1-LC PHWWP	24	0.901	+2
Caerin 1.1-LC 11K.19K.PKAVP	24	0.506	+5
Caerin 1.1-LC 7.11.19K.V(D)V(D)	24	0.550	+5
Caerin 1.1-LC PKAL(D)P	24	0.526	+6

Hydrophobicity *: hydrophobicity of peptide was predicted with HeliQuest program (https://heliquest.ipmc.cnrs.fr/index.html, accessed on 4 November 2025).

**Table 3:** MICs (µM) and MBCs (µM) of Caerin 1.1-LC and analogues against Gram-positive bacteria and Gram-negative bacteria.

Peptide	Gram-Negative Bacteria Strains							Gram-Positive Bacteria Strains		
	*E. coli* (ATCC 8739)	*E. coli* (ATCC BAA-2340)	*E. coli* (NCTC 13846)	*K. pneumoniae* (ATCC 43816)	*K. pneumoniae* (ATCC BAA-2342)	*P. aeruginosa* (ATCC 9027)	*A. baumannii* (ATCC BAA-747)	*S. aureus* (ATCC 6538)	*E. faecalis* (NCTC 12697)	MRSA (NCTC 12493)
Caerin 1.1-LC	16/64	8/16	4/32	16/64	16/64	64/128	16/128	64/128	>128	32/128
Caerin 1.1-LC 11K	8/16	2/8	4/8	16/32	8/16	16/32	32/32	8/32	128/128	8/8
Caerin 1.1-LC 11K.22K	1/2	2/2	4/8	8/16	4/4	4/8	4/16	4/16	64/64	4/8
Caerin 1.1-LC 11K.12K.12W	2/2	2/4	2/4	2/4	8/8	2/4	4/4	1/2	32/32	1/16
Caerin 1.1-LC 11K.12K.19W	2/2	2/2	2/4	4/4	4/4	4/4	4/4	2/2	16/32	2/2
Caerin 1.1-LC 7.11.19K	2/2	2/2	4/4	4/4	4/4	4/4	4/8	4/4	128/128	8/8
Caerin 1.1-LC 2P-2A	>128	>128	>128	>128	>128	>128	>128	>128	>128	>128
Caerin 1.1-LC PGGGP	128/128	128/>128	128	>128	>128	>128	>128	>128	>128	>128
Caerin 1.1-LC PRVVP	2/8	2/4	4/16	8/16	8/32	8/8	32/32	8/16	>128	4/8
Caerin 1.1-LC PKVVP	4/8	2/4	2/4	16/32	8/32	8/32	32/32	16/32	>128	8/8
Caerin 1.1-LC PHLLP	4/8	4/4	4/4	8/8	8/16	32/64	4/8	4/4	32/32	4/4
Caerin 1.1-LC PHIIP	4/8	4/8	4/8	16/16	8/16	32/64	8/8	4/8	>128	4/4
Caerin 1.1-LC PHWWP	32/64	16/32	64/128	>128	>128	>128	>128	>128	>128	>128
Caerin 1.1-LC 11K.19K.PKAVP	8/8	8/8	2/2	64/64	>128	32/64	128/128	>128	>128	64/64
Caerin 1.1-LC 7.11.19K.V(D)V(D)	2/4	4/4	8/8	64/128	16/32	4/4	32/128	128/128	>128	64/64
Caerin 1.1-LC PKAL(D)P	2/2	2/2	8/8	4/4	4/4	4/4	32/32	32/32	>128	4/8

**Table 4:** HC10 (μM), GM _MIC_ (μM), and TI values of Caerin 1.1-LC and its analogues.

Peptides	HC_10_	GM _MIC_ * Gram-Negative	GM _MIC_ * Gram-Positive	TI * Gram-Negative	TI * Gram-Positive
Caerin 1.1-LC	37.5	80.63	14.25	0.47	2.63
Caerin 1.1-LC 11K	35.8	8.8	20.1	4.06	1.78
Caerin 1.1-LC 11K.22K	10.3	10.08	3.17	1.02	3.25
Caerin 1.1-LC 11K.12K.12W	8.5	3.17	2	2.68	4.25
Caerin 1.1-LC 11K.12K.19W	10.5	4	2.97	2.62	3.53
Caerin 1.1-LC 7.11.19K	46.5	16	3.28	2.91	14.18
Caerin 1.1-LC 2P-2A	256	256	256	1	1
Caerin 1.1-LC PGGGP	256	190.21	256	1.34	1
Caerin 1.1-LC PRVVP	21.2	20.16	5.94	1.05	3.57
Caerin 1.1-LC PKVVP	25.8	32	12.14	0.81	2.12
Caerin 1.1-LC PHLLP	1.6	8	6.56	0.2	0.244
Caerin 1.1-LC PHIIP	2.4	16	8	0.15	0.3
Caerin 1.1-LC PHWWP	256	105	256	2.44	1
Caerin 1.1-LC 11K.19K.PKAVP	256	26.08	161.5	9.82	1.59
Caerin 1.1-LC 7.11.19K.V(D)V(D)	256	9.75	128	26.26	2
Caerin 1.1-LC PKAL(D)P	43.4	4.87	64	8.91	0.68

* GM _MIC_: geometric mean of MICs, which is calculated using an online calculator (https://www.alcula.com/calculators/statistics/geometric-mean/, accessed on 4 November 2025). * TI: therapeutic index, calculated as HC10/GM _MIC_. HC10 represents the haemolysis concentration at which a peptide induces 10% haemolysis of horse erythrocytes. If the peptide exhibited no haemolysis effect at maximum testing concentration (128 μM), HC10 was recorded as 256 μM.

**Table 5:** IC_50_ (μM) of Caerin 1.1-LC and Caerin 1.1-LC 7.11.19K.V(D)V(D).

Cell Line	IC_50_	
	Caerin 1.1-LC	Caerin 1.1-LC 7.11.19K.V(D)V(D)
MRC-5	14.8	NI *
HEK 293	10.8	129.6
HMEC-1	79.3	NI
HaCaT	61.2	NI
RAW 264.7	6.0	NI

NI *, no inhibition activity.

**Table 6:** MIC (μM) of Caerin 1.1-LC and analogues against *E. coli* (ATCC-BAA 2340) under different environments.

Peptide	MIC (μM)							
	Na^+^	K^+^	NH_4_^+^	Mg^2+^	Ca^2+^	Fe^3+^	FBS	Control
Caerin 1.1-LC	16	16	16	32	64	16	32	16
Caerin 1.1-LC 11K.22K	8	8	8	16	32	8	16	4
Caerin 1.1-LC 11K.12K.12W	4	4	4	8	32	4	8	4
Caerin 1.1-LC 11K.12K.19W	4	4	4	4	8	4	8	4
Caerin 1.1-LC 7.11.19K	2	2	2	4	64	2	16	2
Caerin 1.1-LC PKAL(D)P	4	2	2	16	128	2	16	2
Caerin 1.1-LC 7.11.19K.V(D)V(D)	16	4	4	32	128	4	16	4

## Data Availability

The data that support the findings of this study are available on request from the corresponding author.

## Supplementary Materials

The following supporting information can be downloaded at: https://www.mdpi.com/article/10.3390/pharmaceutics17111500/s1. Figure S1: The RP-HPLC chromatogram and mass spectrum of peptide Caerin 1.1-LC and its analogues
