# Supplementary material for: Serendipitous Hinge Modulation Hypothetically Reprograms Caerin 1.1-LC Antibacterial Mechanism and Gram-Negative Selectivity
Source: Pharmaceutics. 2025 Nov 20;17(11):1500. doi: 10.3390/pharmaceutics17111500 (PMC12655529; doi:10.3390/pharmaceutics17111500)
Supplement: Supplementary file 1 [file pharmaceutics-17-01500-s001.zip › pharmaceutics-3971647-supplementary.pdf]

pharmaceutics

MDPI

Supplementary Materials: Serendipitous Hinge Modulation Hypothetically Reprograms Caerin 1.1-LC Antibacterial Mechanism and Gram-Negative Selectivity

Zhengze Sun, Ruixin Zhao, Yueao Zhang, Xiaonan Ma, Yangyang Jiang, Tao Wang, Xiaoling Chen, Chengbang Ma, Tianbao Chen, Chris Shaw, Mei Zhou and Lei Wang

Caerin 1.1-LC

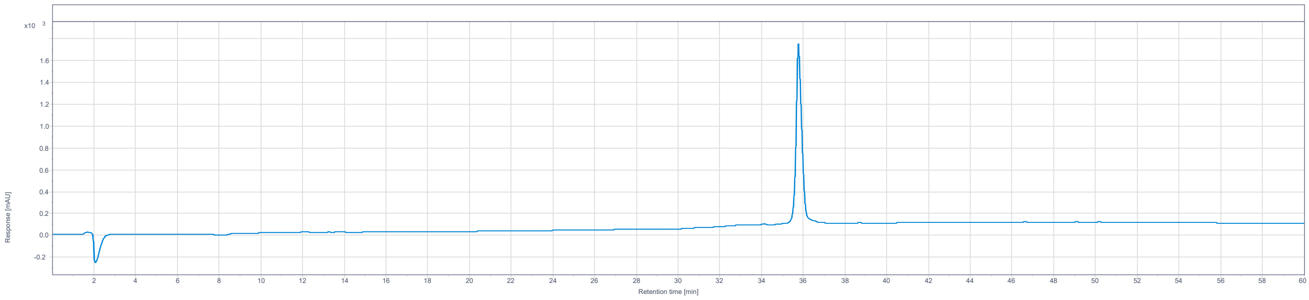

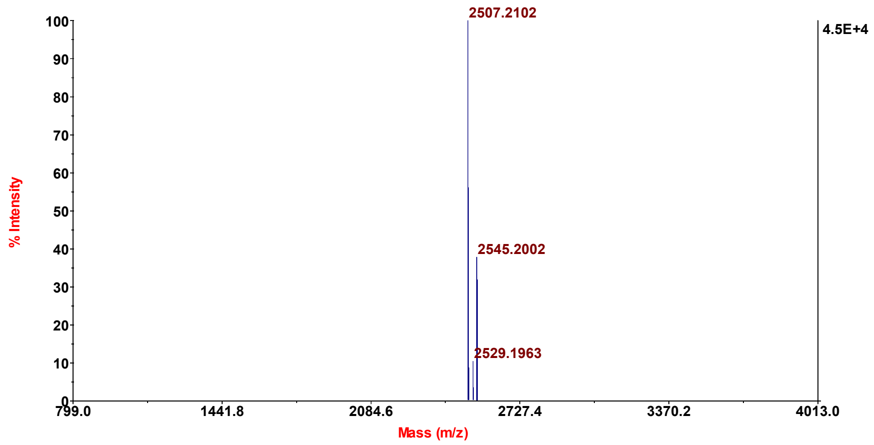

Caerin 1.1-LC-11K

 https://doi.org/10.3390/xxxxx

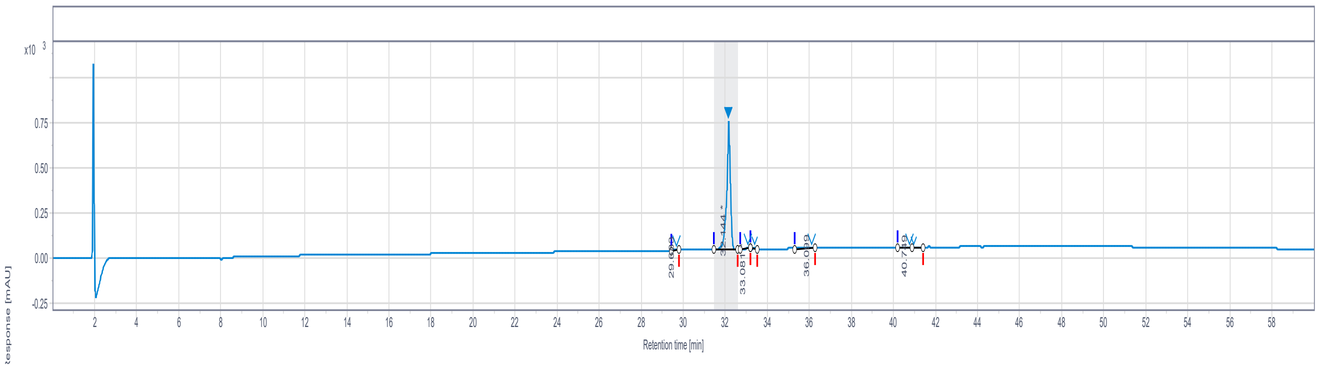

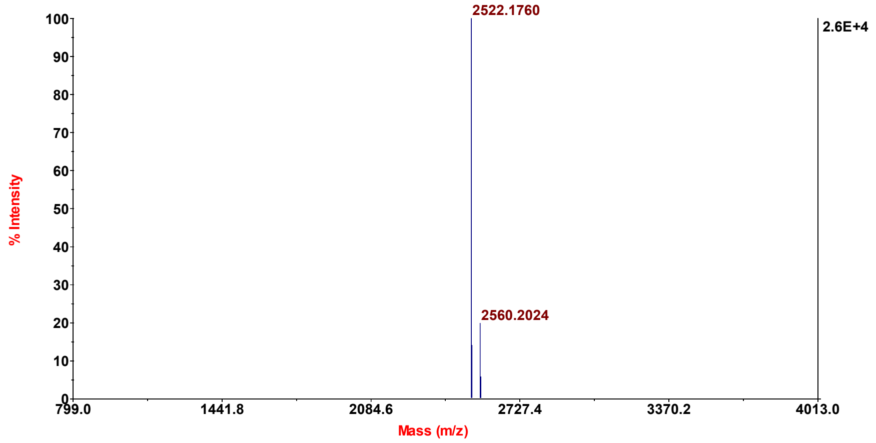

Caerin 1.1-LC-11K.22K

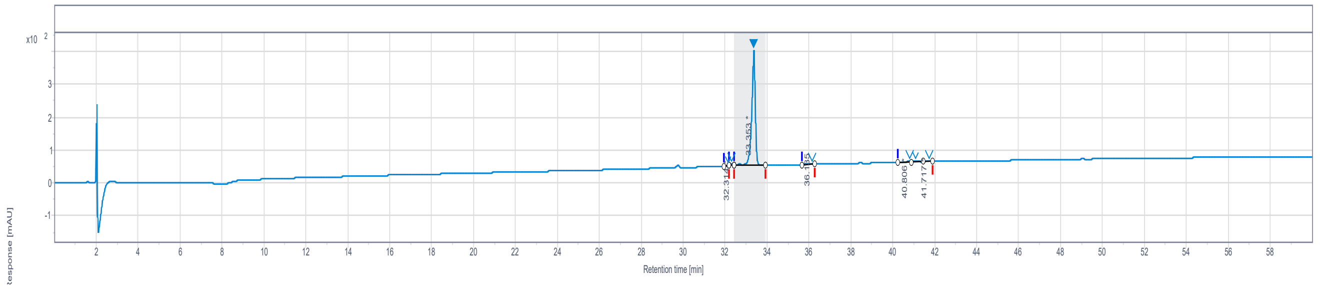

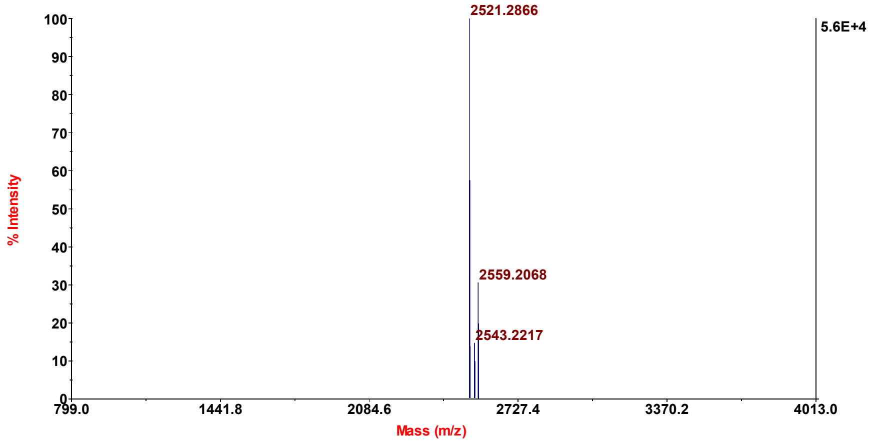

Caerin 1.1-LC-11K.22K.12W

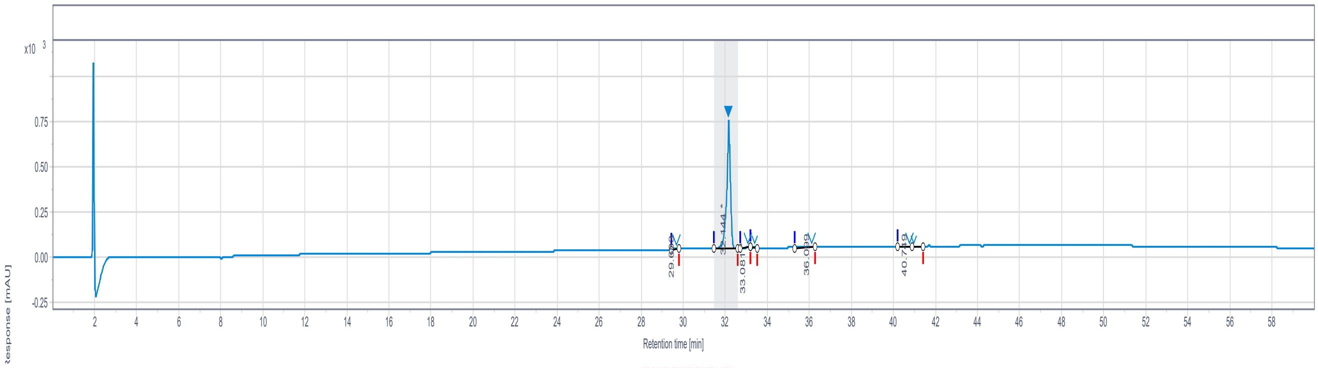

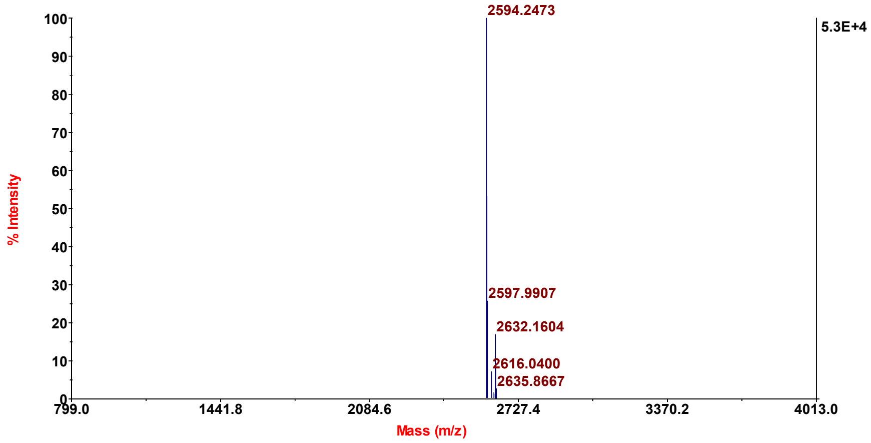

Caerin 1.1-LC-11K.22K.19W

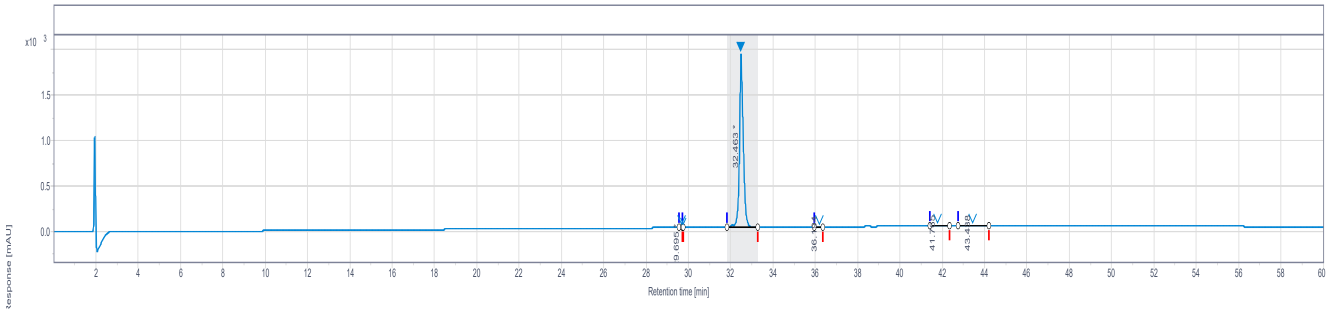

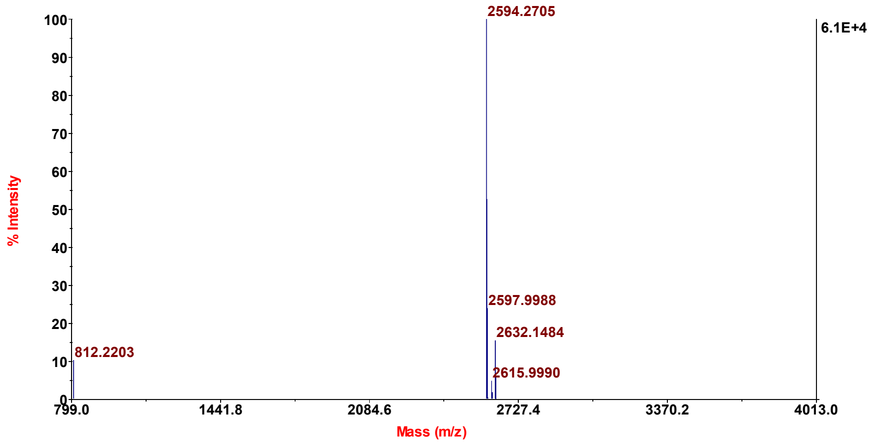

Caerin 1.1-LC 7.11.19K

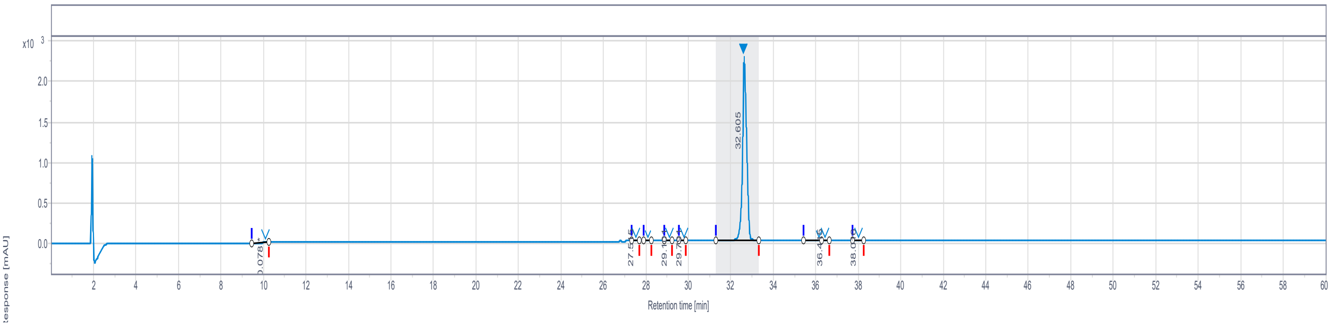

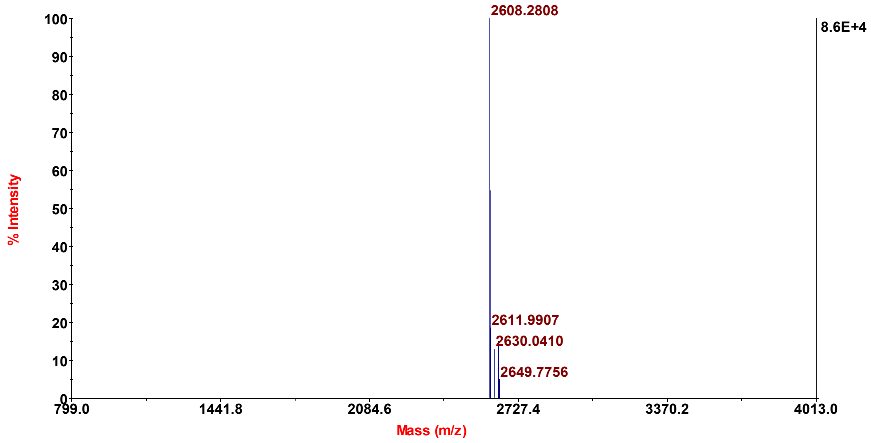

Caerin 1.1-LC 2P-2A

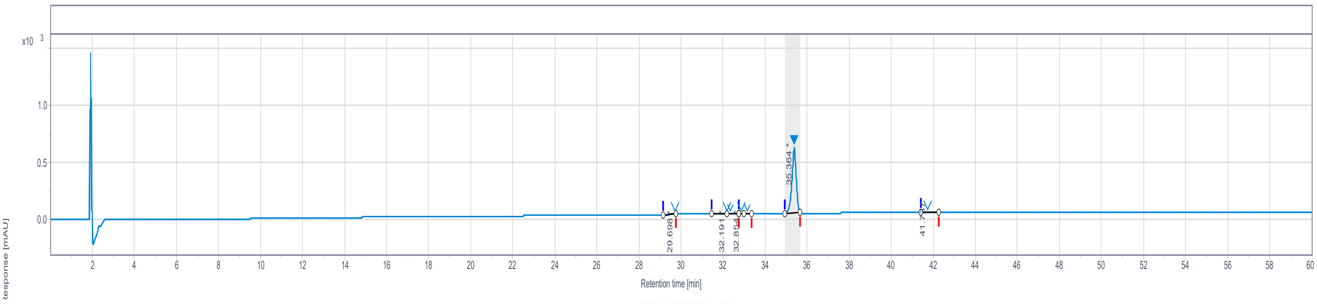

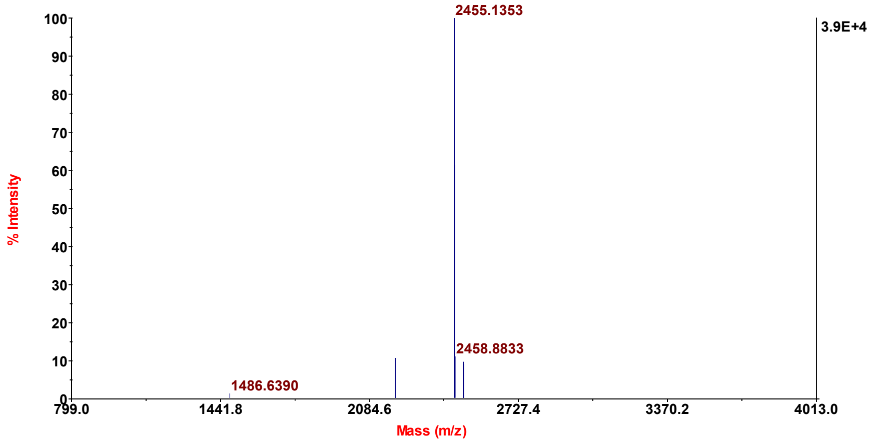

Caerin 1.1-LC PGGGP

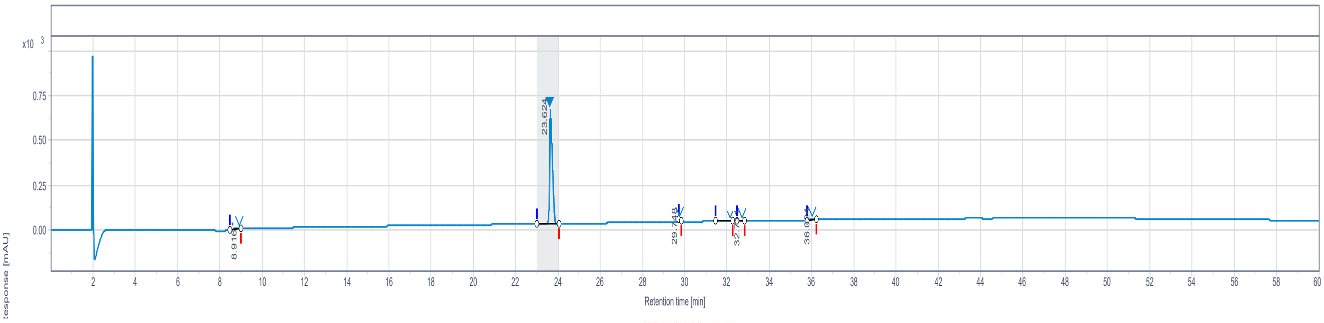

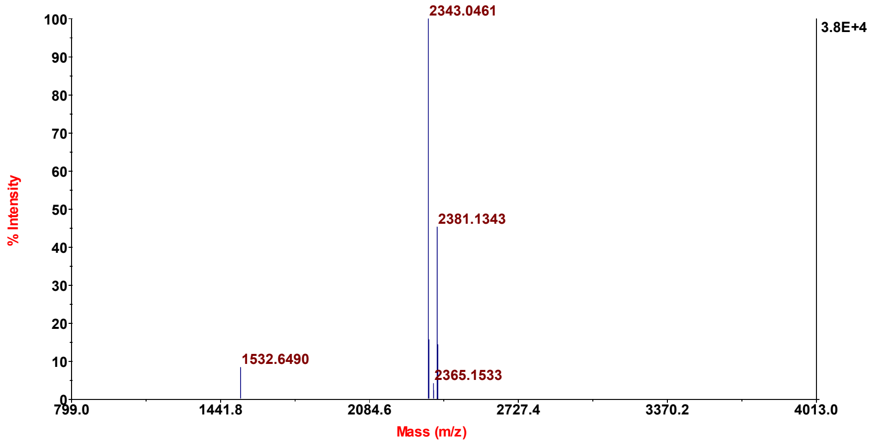

Caerin 1.1-LC PRVVP

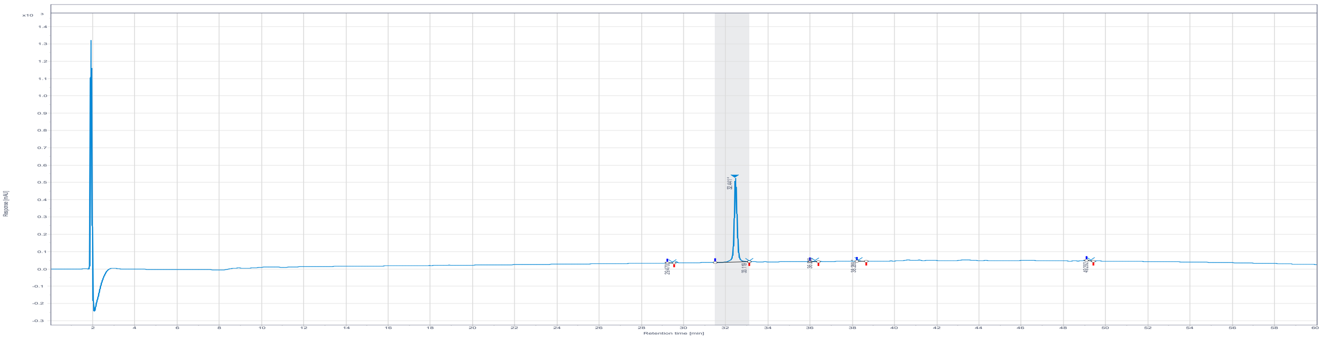

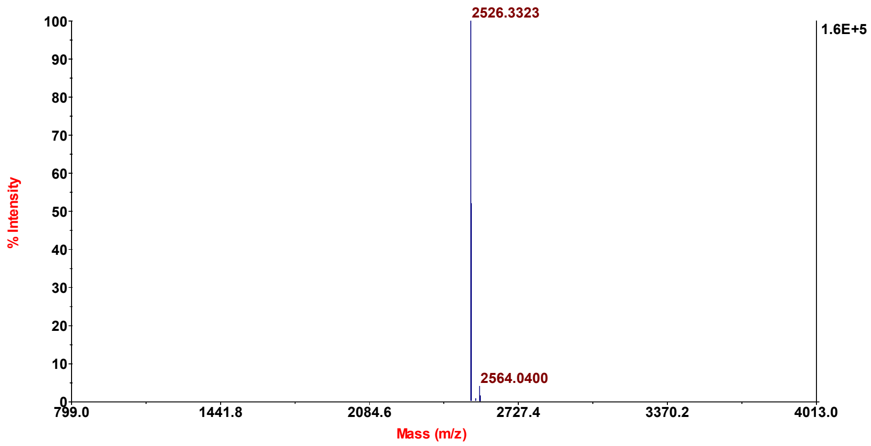

Caerin 1.1-LC PKVVP

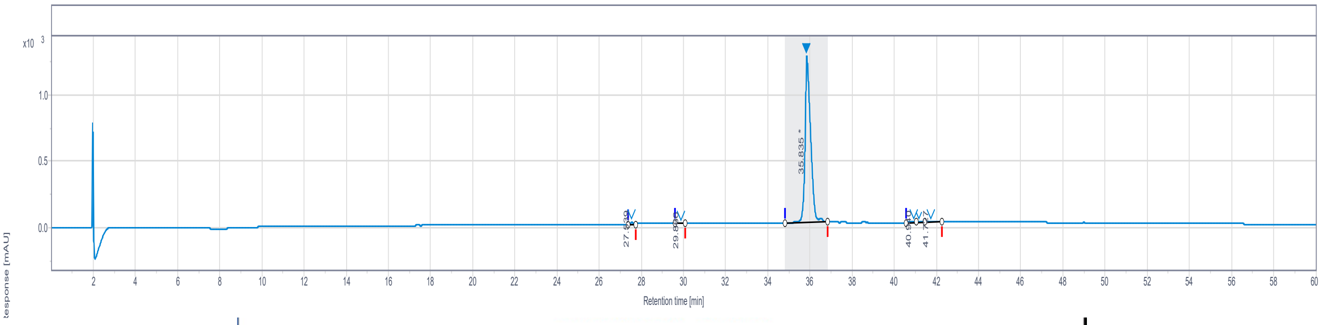

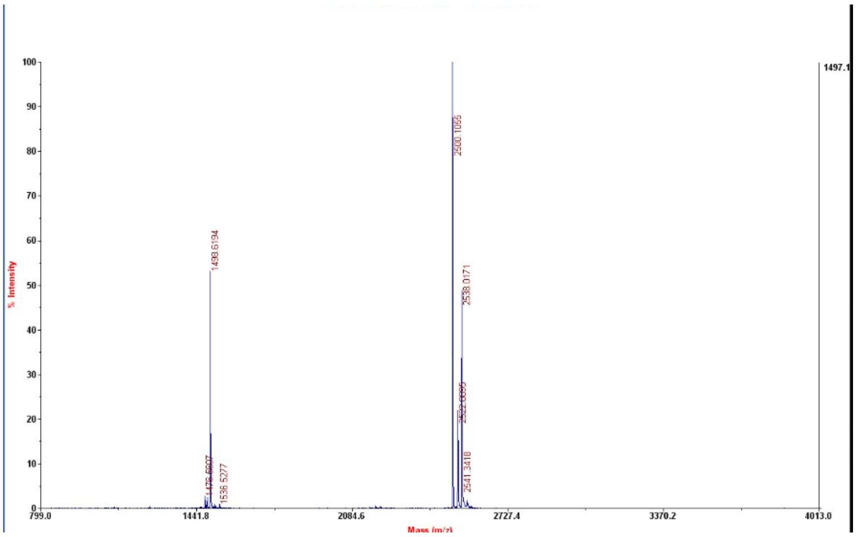

Caerin 1.1-LC PHLLP

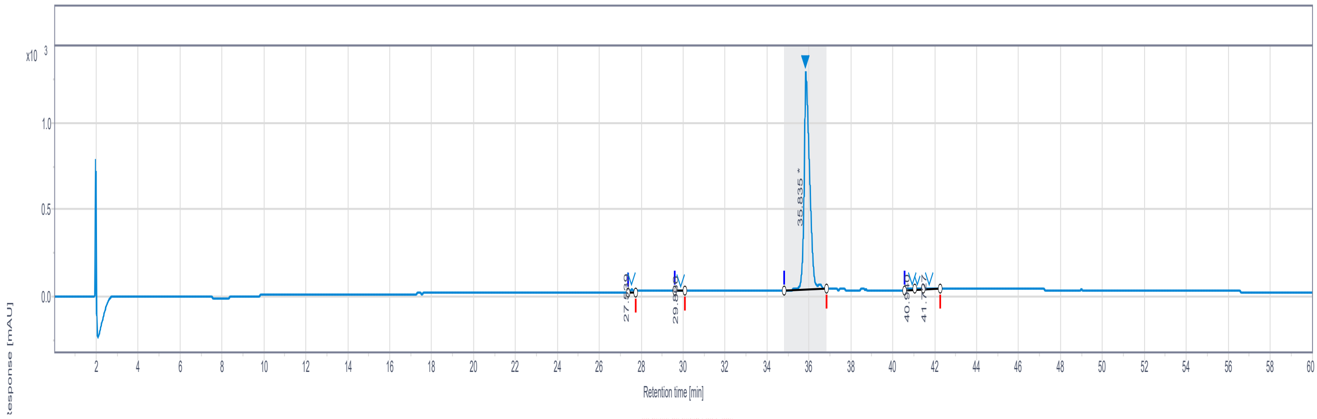

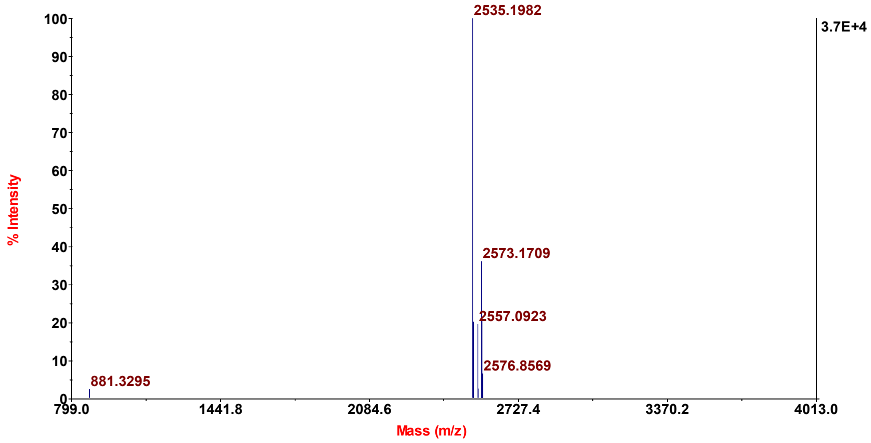

Caerin 1.1-LC PHIIP

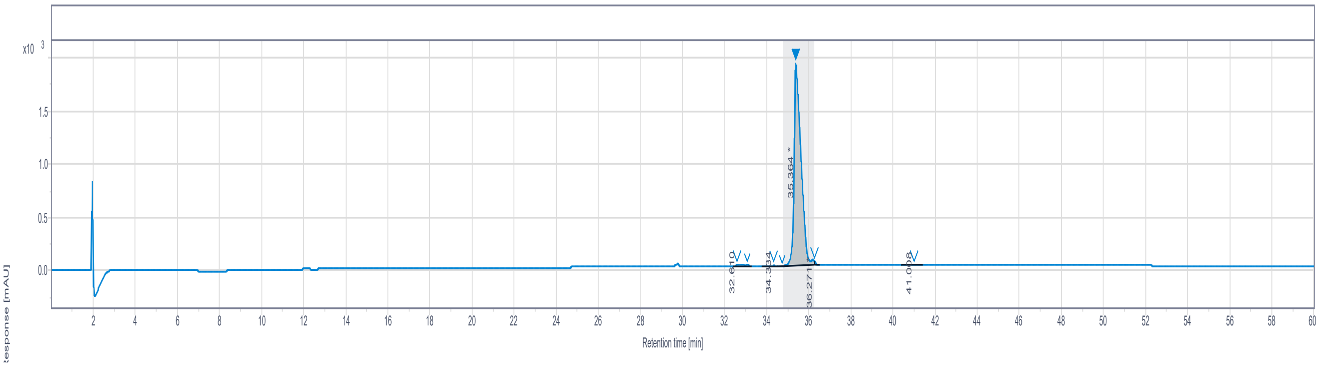

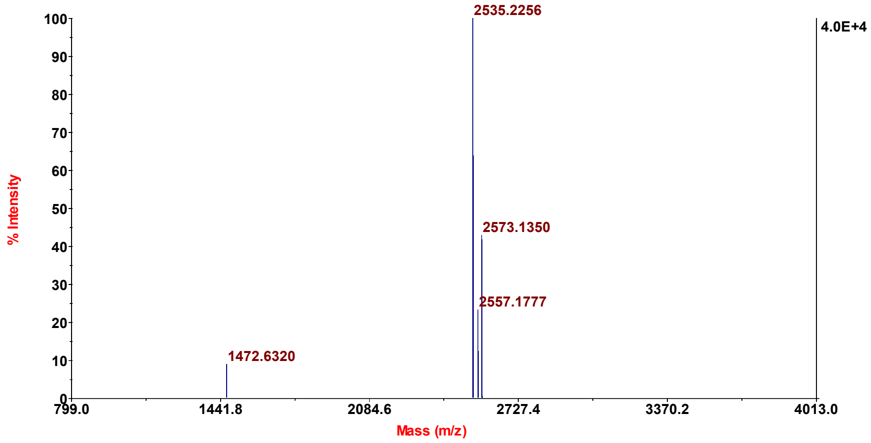

Caerin 1.1-LC PHWWP

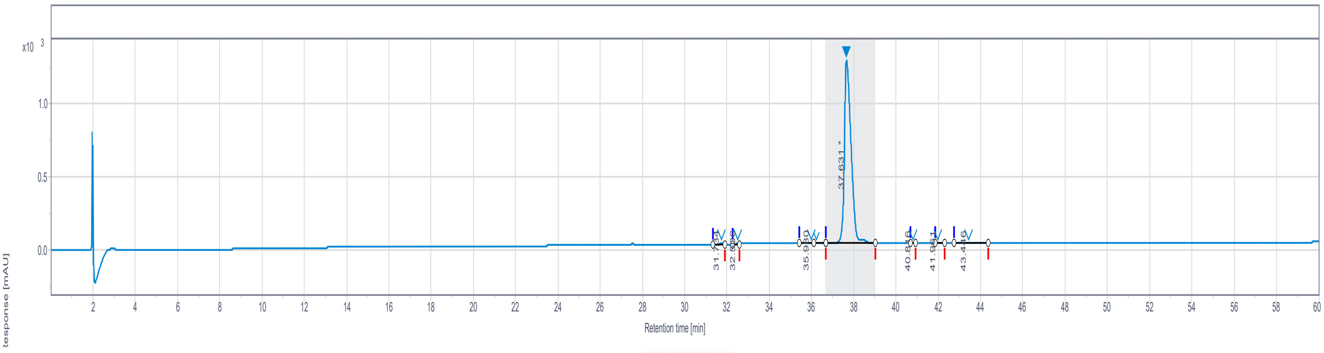

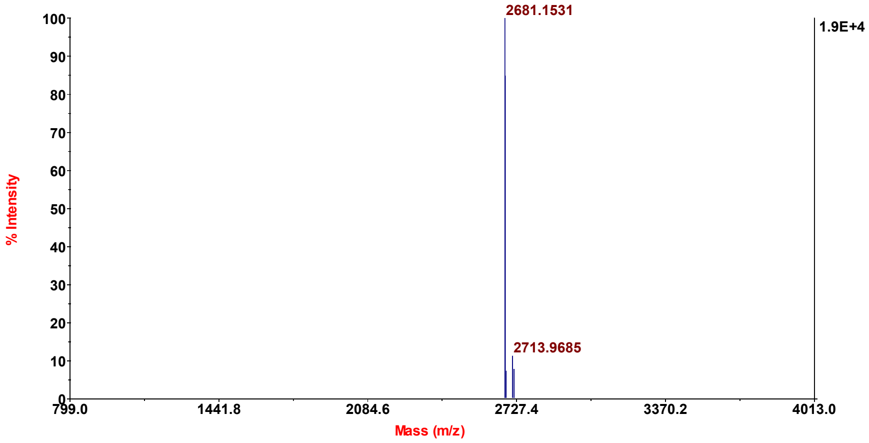

Caerin 1.1-LC 11K.19K.PKAVP

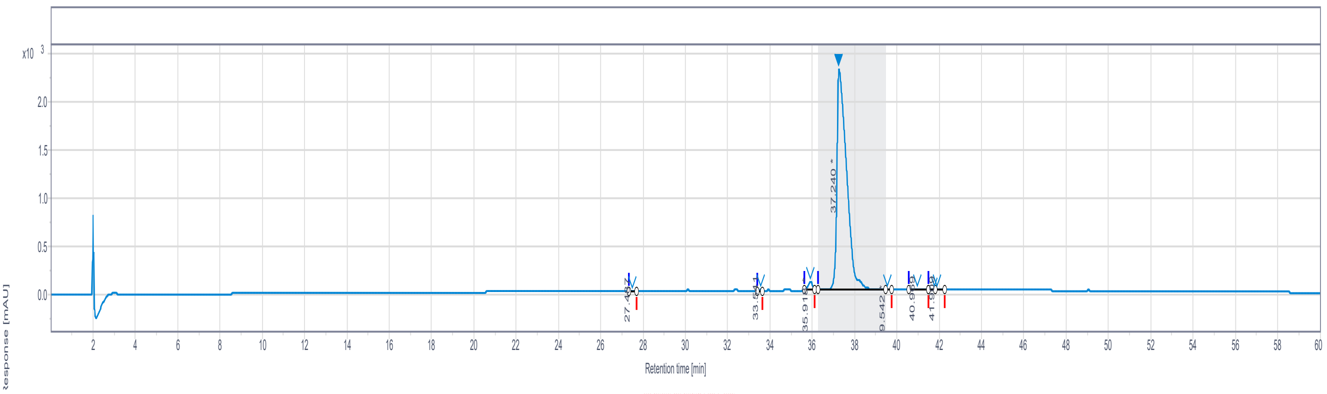

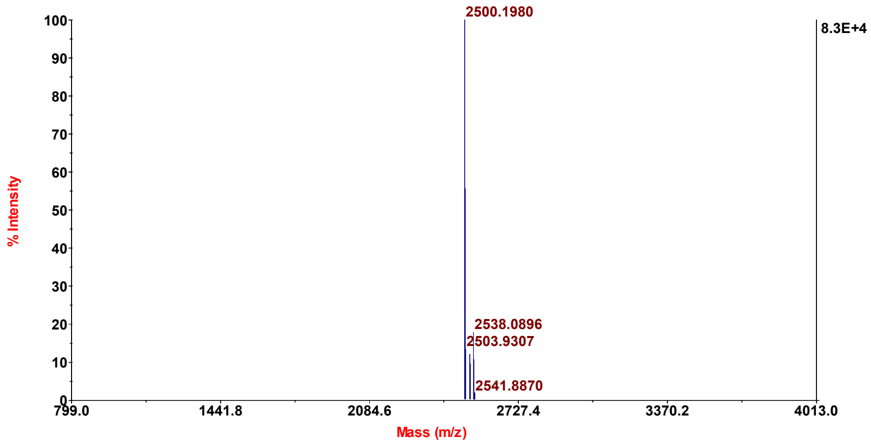

Caerin 1.1-LC 7.9.11K.V(D)V(D)

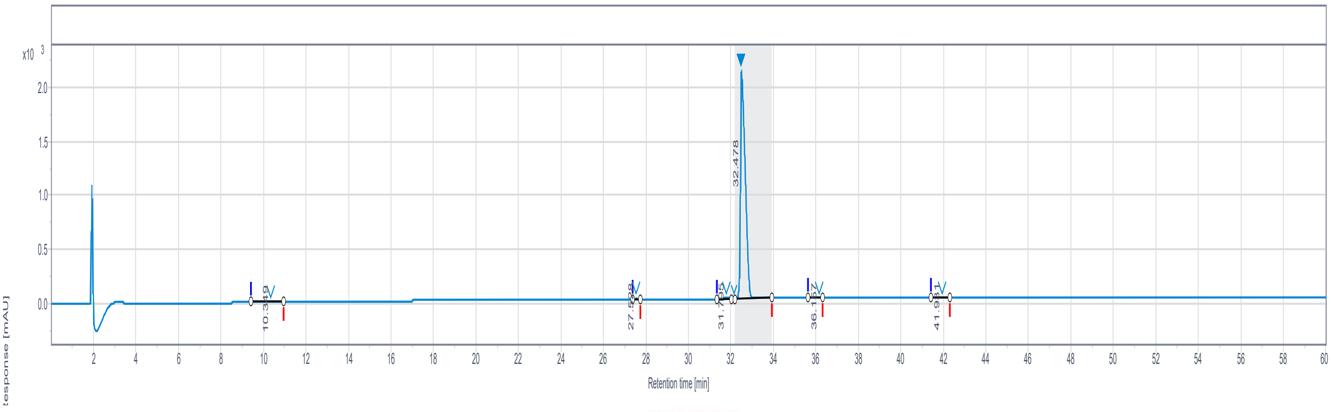

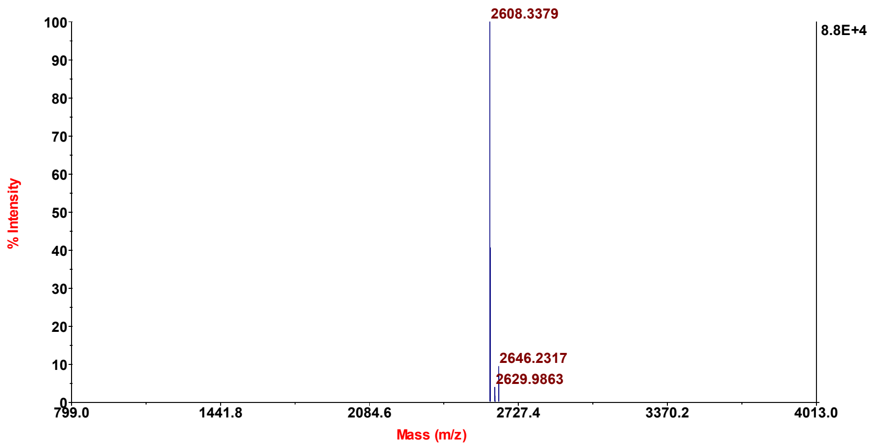

Caerin 1.1-LC PKAL(D)P

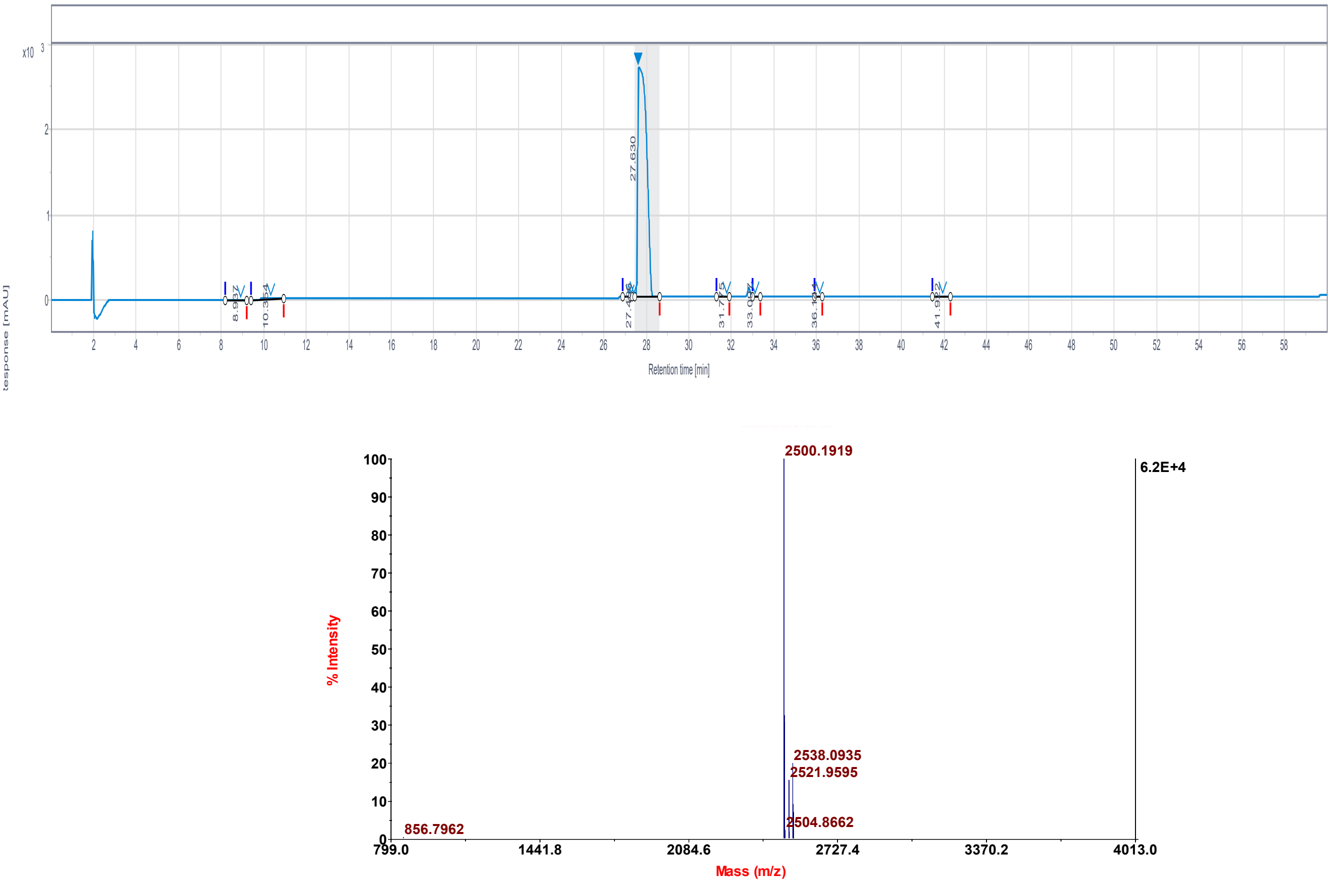

Figure S1. The RP-HPLC chromatogram and mass spectrum of peptide Caerin 1.1-LC and its analogues.
